# Effects of Low-Carbohydrate and Ketogenic Diets on Anaerobic Performance in Competitive Athletes: A Systematic Review and Meta-Analysis

**DOI:** 10.3390/nu18101589

**Published:** 2026-05-16

**Authors:** Mateusz Gawelczyk, Jakub Chycki, Adam Maszczyk, Adam Zając

**Affiliations:** Institute of Sport Sciences, Academy of Physical Education, 40-065 Katowice, Poland; m.gawelczyk@awf.katowice.pl (M.G.); j.chycki@awf.katowice.pl (J.C.); a.maszczyk@awf.katowice.pl (A.M.)

**Keywords:** low-carbohydrate diet, ketogenic diet, anaerobic performance, sprint performance, sports nutrition, fat oxidation, trained athletes

## Abstract

Background/Objectives: Low-carbohydrate (LCD) and ketogenic diets (KD) are increasingly adopted by athletes due to their ability to enhance fat oxidation and induce metabolic adaptations. While their effects on aerobic power and capacity have been widely investigated, their influence on anaerobic performance remains unclear. Given the strong dependence of high-intensity exercise on glycolytic metabolism and muscle glycogen availability, carbohydrate restriction may have significant implications for short-duration maximal efforts and repeated high-intensity exercise. Therefore, this systematic review and meta-analysis aimed to evaluate the effects of LCD and KD on anaerobic performance outcomes in trained athletes. Methods: A comprehensive search of five electronic databases (PubMed, SCOPUS, Web of Science, SPORTDiscus, and Cochrane Central Register of Controlled Trials) identified 13 unique studies (yielding 15 study-level entries across three anaerobic performance domains) meeting comprehensive inclusion criteria. Individual study sample sizes ranged from *n* = 5 to *n* = 65 participants, reflecting substantial inter-study variability that should be considered when interpreting pooled estimates. Outcomes included peak and mean power output, repeated sprint performance, blood lactate responses, and markers of substrate utilization. Study quality was assessed using the Newcastle–Ottawa Scale, and meta-analyses were performed using random-effects models where appropriate. Results: Overall, the effects of carbohydrate-restricted diets on anaerobic performance were domain-specific. Some studies reported maintained or slightly improved peak power during single maximal efforts, while others showed no effect. Impairments were more consistently observed in repeated high-intensity exercise. Repeated sprint performance was impaired in several studies, likely reflecting reduced muscle glycogen availability and limited glycolytic ATP production. Carbohydrate restriction consistently increased fat oxidation and was associated with lower blood lactate concentrations during high-intensity exercise. Random-effects meta-analyses yielded domain-specific pooled effect sizes: maintained-to-slightly-improved anaerobic power output (Cohen’s d = +0.29; 95% CI: −0.08 to +0.66), modestly impaired repeated sprint ability (d = −0.33; 95% CI: −0.80 to +0.14), and a large, consistent reduction in blood lactate concentration (d = −0.89; 95% CI: −1.20 to −0.58). Given substantial between-study heterogeneity in intervention durations (2 days to 12 weeks), dietary composition, athlete populations, and outcome measures (1RM, Wingate, CMJ within the power domain; varied protocols within the RSA and lactate domains), these pooled estimates should be interpreted as exploratory rather than confirmatory. Conclusions: LCD and KD appear to have domain-specific effects on anaerobic performance in trained athletes. Although single, short-duration efforts may be preserved in some contexts, repeated, high-intensity performance appears to be more susceptible to impairment. These findings highlight the importance of aligning dietary strategies with the metabolic demands of training and competition.

## 1. Introduction

Dietary macronutrient composition is a key determinant of athletic performance in trained individuals. Traditional sports nutrition paradigms advocate maintaining high carbohydrate availability—typically 6–10 g·kg^−1^·day^−1^—as a key requirement for optimizing performance in both endurance and high-intensity exercise [1,2,3,4].

These recommendations are supported by extensive physiological evidence demonstrating that endogenous muscle glycogen and circulating glucose represent key substrates for rapid adenosine triphosphate (ATP) resynthesis during moderate-to-high intensity exercise, particularly when exercise intensity exceeds ~70% of maximal oxygen uptake (VO_2_max) [5,6,7]. Consequently, contemporary guidelines from major sports nutrition organizations continue to advocate high carbohydrate intake as a cornerstone strategy for supporting training adaptations and maintaining performance during repeated bouts of intense exercise [5,8].

Despite these long-standing recommendations, low-carbohydrate (LCD) and ketogenic diets (KD) have gained increasing popularity among athletes and coaches over the past two decades. These dietary strategies typically restrict carbohydrate intake to ≤130 g·day^−1^ (or <25% of total energy intake) for LCD or <50 g·day^−1^ (or <10% of total energy intake) for KD, thereby promoting metabolic adaptations characterized by increased fat oxidation, elevated circulating ketone bodies, and reduced reliance on carbohydrate metabolism [9,10]. In endurance contexts, these metabolic adaptations have been proposed to enhance metabolic efficiency by increasing the capacity for lipid utilization and reducing glycogen depletion during prolonged exercise [9,10,11]. As a result, ketogenic and low-carbohydrate strategies have been investigated as potential nutritional approaches for improving endurance performance or optimizing body composition in trained athletes.

However, the metabolic requirements of high-intensity exercise differ substantially from those of prolonged endurance activity. In the context of sport, anaerobic performance typically includes short-duration, high-intensity efforts such as sprint performance, repeated sprint ability, maximal power output, and strength-related outcomes. These efforts rely predominantly on phosphocreatine turnover and anaerobic glycolysis for rapid ATP resynthesis [12,13]. During efforts lasting approximately 10–60 s, as well as during repeated high-intensity bouts, glycolytic flux and intramuscular glycogen availability become critical determinants of performance capacity [14,15,16]. Consequently, substantial carbohydrate restriction may reduce the capacity for rapid ATP resynthesis during high-intensity exercise due to limited glycogen availability [13,17,18].

Experimental investigations examining the effects of LCD and KD on anaerobic performance have produced inconsistent findings. Importantly, interpretation of these results depends on whether performance outcomes are expressed in absolute terms (e.g., watts) or relative to body mass (W·kg^−1^), as carbohydrate-restricted diets frequently induce reductions in body mass and fat mass. Some studies have reported impairments in anaerobic capacity following short-term carbohydrate restriction. For example, Wroble et al. observed reductions in peak and mean power output during the Wingate test following a short-term ketogenic diet intervention [19]. Similarly, Michalczyk et al. demonstrated that four weeks of a low-carbohydrate diet significantly reduced total work performed during anaerobic cycling tests in competitive basketball players, although these impairments were reversed following carbohydrate re-loading [20].

In contrast, other studies have reported neutral effects on maximal strength or explosive performance variables. Paoli et al., for example, found no significant differences in vertical jump performance or strength measures in elite gymnasts following a ketogenic diet intervention [21]. Additionally, longer adaptation periods have occasionally been associated with improvements in relative power output, which likely reflect reductions in body mass and fat mass rather than increases in absolute power production [22].

Several factors may contribute to these heterogeneous outcomes, including differences in dietary adaptation duration, metabolic responses, and exercise modality. For example, a four-week ketogenic diet in CrossFit-trained athletes was shown to alter exercise metabolism and substrate utilization without consistently improving high-intensity performance outcomes [22].

First of all, the duration of dietary adaptation appears to play a critical role, as short-term ketogenic interventions are often characterized by glycogen depletion and incomplete metabolic adaptation, whereas longer interventions may allow physiological adjustments that partially compensate for reduced carbohydrate availability [9,23]. Secondly, the specific type of performance assessment may influence the observed outcomes. Explosive efforts, such as jumps and throws, which last only a few seconds, rely predominantly on phosphocreatine stores and are, therefore, less sensitive to carbohydrate availability. In contrast, longer high-intensity efforts involving speed endurance depend more heavily on glycolytic metabolism and may be more susceptible to carbohydrate restriction [12,13].

Finally, changes in body composition associated with carbohydrate restriction may influence relative performance metrics expressed per unit of body mass, complicating interpretation of performance outcomes.

Despite the growing interest in low-carbohydrate and ketogenic diets among athletes, their physiological effects and performance implications remain debated across different exercise modalities and athletic populations, as summarized in recent position stands and reviews [24].

Although several narrative reviews and systematic analyses have examined ketogenic diets in athletic populations broadly, relatively few studies have specifically synthesized evidence regarding their effects on anaerobic performance outcomes in trained athletes. Given the increasing adoption of carbohydrate-restricted dietary strategies in competitive sport and the conflicting findings reported in particular studies, a comprehensive evaluation of the available evidence is warranted. In a previous systematic review and meta-analysis, we examined the effects of low-carbohydrate and ketogenic diets on aerobic performance variables in trained athletes.

However, the metabolic demands of high-intensity exercise differ substantially from those of predominantly aerobic activities. Anaerobic performance relies heavily on rapid ATP resynthesis through phosphocreatine breakdown and glycolysis, processes that depend on adequate carbohydrate availability and intramuscular glycogen stores. Consequently, the extent to which carbohydrate-restricted dietary strategies influence anaerobic performance outcomes in trained athletes remains unclear. Importantly, the interpretation of these effects may be further complicated by the use of ergogenic aids, such as caffeine, which is widely consumed in athletic populations and has been shown to enhance high-intensity and anaerobic performance irrespective of dietary conditions. The inconsistent control or reporting of such co-interventions across studies may, therefore, contribute to variability in observed outcomes.

In the context of this review, anaerobic performance refers to short-duration, high-intensity exercise outcomes including peak and mean power output, sprint performance, repeated sprint ability, and metabolic responses associated with glycolytic energy production. For the purpose of this review, dietary interventions were classified based on carbohydrate content. A low-carbohydrate diet (LCD) was defined as a diet providing ≤25% of total energy from carbohydrates. A ketogenic diet (KD) was defined as a more restrictive form of carbohydrate reduction, typically providing ≤10% of total energy or ≤50 g of carbohydrates per day, sufficient to induce nutritional ketosis.

Therefore, the aim of the present systematic review and meta-analysis was to evaluate the effects of low-carbohydrate and ketogenic dietary interventions on anaerobic performance variables in competitive athletes. Specifically, this review synthesizes evidence from controlled studies assessing outcomes such as peak and mean power output, sprint performance, repeated sprint ability, strength measures, and other indices of high-intensity exercise capacity.

## 2. Materials and Methods

### 2.1. Study Design and Search Strategy

A comprehensive two-phase literature retrieval strategy was implemented to maximize both reproducibility and recall, in accordance with PRISMA 2020 recommendations.

Phase 1—Conventional Boolean searches (primary, fully reproducible). Database-specific Boolean searches were conducted in five major bibliographic databases: PubMed/MEDLINE, Scopus, Web of Science, SPORTDiscus (EBSCO), and the Cochrane Central Register of Controlled Trials. The full PubMed/MEDLINE search strategy, including all Medical Subject Headings (MeSH), free-text title/abstract terms, Boolean operators, filters, and the date of search, is reported in Supplementary Table S3, Section A; the corresponding syntactic adaptations preserving semantic equivalence in Scopus (TITLE-ABS-KEY), Web of Science (TS), SPORTDiscus (AB/TI), and Cochrane CENTRAL (ti,ab,kw) are reported in Supplementary Table S3, Section B. Each query combined four conceptual blocks linked with the Boolean operator AND: (i) carbohydrate-restriction terms (“ketogenic diet” OR “low-carbohydrate” OR “LCHF” OR “low-CHO” OR related variants); (ii) anaerobic-performance terms (“athletic performance” OR Wingate OR sprint OR “peak power” OR “mean power” OR “anaerobic capacity” OR “repeated sprint ability” OR RSA OR “countermovement jump” OR CMJ OR “1RM” OR “blood lactate”); (iii) population terms (athlete* OR trained OR elite OR competitive); and (iv) study-design terms (random* OR crossover OR “controlled trial” OR intervention*). Searches were restricted to peer-reviewed publications in English, conducted through October 2025, and re-executable from the strings reported in Supplementary Table S3.

Phase 2—Semantic supplementary search (recall enhancement). To complement the Boolean searches and capture conceptually relevant studies that strict keyword matching may have missed—including methodologically heterogeneous low-carbohydrate/ketogenic intervention trials that authors had described using non-standard terminology—a semantic search was additionally conducted with the Elicit research platform (Elicit, Inc.; elicit.com), which queries approximately 138 million records indexed in Semantic Scholar and OpenAlex, using language-model-based relevance ranking rather than exact-string matching. The 500-record output reflects the maximum number of ranked results that the Elicit interface returns per query (a tool-imposed ceiling, not an arbitrarily chosen user threshold), which we accepted in full to maximize recall at the screening stage. The semantic query employed the same four conceptual blocks as the Boolean strategy. The grey-literature and preprint-server component (bioRxiv, medRxiv) was likewise screened through the Elicit interface, as detailed in Supplementary Table S3, Section C; preprints were retained at the screening stage only for the purpose of identifying potentially eligible studies, and only records that had achieved peer-reviewed publication status by the time of full-text assessment met the inclusion criteria for the final synthesis (Section 2.2.5). To further enhance recall and reduce dependency on either retrieval method alone, the reference lists of all included studies and of relevant prior reviews were manually back-screened by two reviewers working independently.

**Figure 1:**
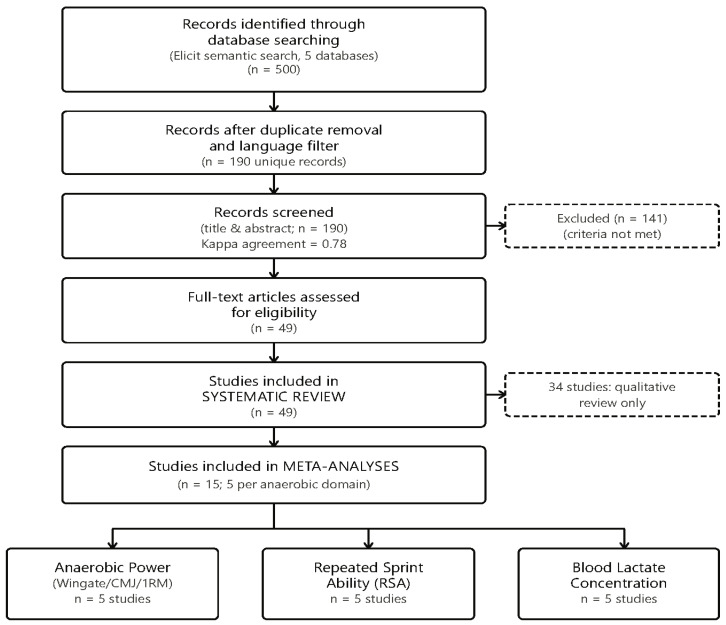
PRISMA 2020 Flow Diagram illustrating the study selection process. Records were identified through a two-phase search strategy combining database-specific Boolean searches across five major bibliographic databases (PubMed/MEDLINE, Scopus, Web of Science, SPORTDiscus, Cochrane CENTRAL) with a supplementary semantic search of Semantic Scholar/OpenAlex and grey-literature screening of bioRxiv/medRxiv via the Elicit platform (*n* = 500 unique records after merging across sources; full search strategies in Supplementary Table S3). Following duplicate removal and language filtering, 190 unique records underwent title and abstract screening (κ = 0.78). After full-text assessment of 49 articles, 13 unique studies (yielding 15 study-level entries; 5 per anaerobic domain: anaerobic power, repeated sprint ability, and blood lactate concentration) met inclusion criteria; the remaining 36 articles were excluded at the full-text stage.

Records retrieved from both phases were merged into a single bibliographic library, automatically duplicated, and language-filtered prior to title-and-abstract screening; the combined initial pool, deduplication, and downstream selection counts are reported in Section 2.3 and the PRISMA 2020 flow diagram (Figure 1). A completed PRISMA 2020 checklist is provided as Supplementary Table S1; the prospective protocol was registered in PROSPERO (CRD420261277181, registered title: “Effects of Low-Carbohydrate and Ketogenic Diets on Aerobic and Anaerobic Performance in Trained Athletes: A Systematic Review and Meta-Analysis”), encompassing both aerobic and anaerobic performance outcomes; the present manuscript reports the anaerobic outcomes, whereas the aerobic outcomes are reported in a companion publication.

### 2.2. Eligibility Criteria and Study Selection

#### 2.2.1. Population

Studies were included if they enrolled competitive athletes, defined as individuals with a history of regular structured training and participation in organized sport. A minimum threshold of ≥6 months of structured training was applied as an initial eligibility criterion; however, in practice, the included studies predominantly involved athletes with substantially longer training histories (typically ≥2–3 years), consistent with competitive or highly trained populations.

The age range was restricted to 18–45 years to ensure applicability to adult populations. Minimum baseline characteristics included documented competitive participation and training consistent with structured sport preparation. This approach aligns with established athlete classification frameworks (e.g., McKay et al. [25]), which consider training history, performance level, and competition experience when distinguishing trained and competitive individuals from recreational populations.

Studies enrolling untrained individuals, recreational athletes, or sedentary populations were excluded. Studies including mixed populations (trained and untrained) were excluded if separate data for trained subgroups could not be extracted.

#### 2.2.2. Intervention

Studies were included if they examined low-carbohydrate dietary interventions defined as ≤130 g·day^−1^ total carbohydrate intake or ≤25% energy from carbohydrates, or ketogenic diet interventions defined as <50 g carbohydrates·day^−1^ or <10% energy from carbohydrates with confirmed physiological ketosis (β-hydroxybutyrate ≥0.5 mmol·L^−1^). Intervention duration required ≥3 days for acute dietary manipulations or ≥4 weeks for chronic adaptation protocols. Studies documenting dietary compliance through objective measures (food diaries, urinary ketones, serum ketone bodies, macronutrient analysis) or well-documented adherence protocols were included. Studies involving supplementation combined with dietary intervention, training modifications concurrent with diet manipulation, or other confounding co-interventions were excluded if dietary effects could not be isolated.

#### 2.2.3. Comparison

Studies were included if they incorporated appropriate control or comparison conditions including high-carbohydrate diets (>60% energy from carbohydrates or >300 g·day^−1^), balanced/mixed diets (45–55% energy from carbohydrates), or habitual dietary intake. Studies employing within-subject comparisons (cross-over designs) comparing identical individuals under low-carbohydrate and control conditions were included. Studies without explicit control groups were excluded.

#### 2.2.4. Outcomes

Studies were included if they measured minimum two variables encompassing the following domains. Anaerobic performance variables encompassed Wingate test outcomes including peak power (W, W·kg^−1^), mean power (W, W·kg^−1^), and total work (J, J·kg^−1^), repeated sprint ability (RSA) measured through multiple maximal sprints, one-repetition maximum (1RM) strength in various lifting movements, vertical jump or countermovement jump (CMJ) height measured in cm, and sprint power during time trials or sport-specific tests. Metabolic variables included blood lactate concentration (mmol·L^−1^) measured at rest or peak exercise intensity, base excess (mmol·L^−1^) or acid-base balance variables.

#### 2.2.5. Study Design

Eligible study designs included randomized controlled trials (RCT), randomized cross-over trials, parallel group designs, and repeated-measures cross-over protocols. Minimum methodological requirements included: (1) prospective study design; (2) randomized allocation to intervention sequence (for cross-over designs); (3) appropriate washout periods (≥48 h) between intervention conditions in cross-over designs; (4) quantifiable outcome data reported as means and standard deviations or means with 95% confidence intervals; (5) peer-review publication status; and (6) sufficient methodological detail for quality assessment.

Studies were excluded if they: (1) enrolled untrained or recreationally active individuals; (2) lacked control or comparison group; (3) provided no confirmation of dietary adherence; (4) included animal studies, case reports, or conference abstracts without subsequent peer-reviewed publication; (5) combined dietary intervention with confounding nutritional supplements, co-prescribed medications, or concurrent training modifications that prevented isolation of dietary effects; (6) lacked complete numerical data (means ± SD or 95% CI) necessary for meta-analysis; or (7) were published in languages other than English.

### 2.3. Study Selection Process

The combined two-phase literature search (database-specific Boolean searches across PubMed/MEDLINE, Scopus, Web of Science, SPORTDiscus, and Cochrane Central Register of Controlled Trials, supplemented by a semantic search of Semantic Scholar/OpenAlex and grey-literature screening of bioRxiv and medRxiv via the Elicit platform; full strategies in Supplementary Table S3) identified 500 unique records after merging across sources. After removal of duplicates and application of automated filtering tools based on language and publication type, 190 unique records remained for title and abstract screening. Title and abstract screening were performed by two independent reviewers (Kappa agreement = 0.78) using standardized criteria covering population, intervention, study design, comparator/control condition, performance outcomes, study duration, publication quality, dietary compliance, and absence of confounding co-interventions. Reviewers applied a holistic screening approach, integrating all criteria simultaneously rather than using sequential threshold-based filtering. Forty-nine records were identified as potentially meeting eligibility criteria and were retrieved for full-text assessment. Full-text eligibility assessment and data extraction were conducted independently by two reviewers. Any disagreements were resolved through discussion, and when consensus could not be reached, a third reviewer was consulted.

Of the 49 full-text articles assessed, 13 unique studies met the comprehensive inclusion criteria for anaerobic performance outcomes in trained athletes and were incorporated into the final systematic review and meta-analysis. These 13 studies represent those excluded from the companion aerobic-focused review because their primary outcomes were anaerobic performance variables: Wingate test outcomes and sprint power, one-repetition maximum strength, countermovement jump height, repeated sprint ability, and blood lactate concentration during high-intensity efforts. Two of the 13 included studies—McKay et al. [26] and Prins et al. [27], reported relevant outcomes in two distinct anaerobic performance domains and, therefore, contributed data to two of the three meta-analyses; the remaining 11 studies each contributed to a single domain. Treating each study × domain combination as one analytic unit, this yielded 15 study-level entries distributed evenly across the three primary meta-analyses (5 entries per domain). Throughout the manuscript, “13 included studies” refers to the unique primary studies, whereas “15 study-level entries” refers to the analytic units used in the pooled meta-analyses; this distinction is applied consistently in all tables, figures, and the PRISMA flow diagram. The complete selection process is illustrated in Figure 1.

### 2.4. Data Extraction and Quality Assessment

Two independent reviewers conducted data extraction using standardized data collection forms developed a priori. Extracted variables included: (1) study identification: author names, publication year, study design classification, and funding sources of the included studies; (2) population characteristics: sample size (total and per group), participant demographics (age range, mean ± SD), training status/sport discipline, baseline fitness measures; (3) intervention characteristics: dietary type (low-carbohydrate vs. ketogenic), carbohydrate restriction magnitude (g·day^−1^ or % energy), fat percentage, protein percentage, total energy intake, duration of intervention, dietary compliance verification methods; (4) comparison/control diet composition and duration; (5) study methodology: design (crossover, parallel, RCT), washout period duration (if applicable), randomization methods, blinding status; (6) performance outcome measures: specific variables measured, exercise protocols (intensity, duration, modality, equipment), timing of measurements relative to dietary intervention; (7) key results: baseline and post-intervention values for all measured variables, effect sizes or percentage changes, statistical significance (*p*-values, 95% confidence intervals), direction of effect (improvement, impairment, no change); (8) adaptation timeline: duration of dietary intervention prior to performance testing, evidence of metabolic adaptation (ketosis levels, substrate utilization changes), acute versus chronic effect documentation; (9) study quality and limitations: quality scores, potential confounding factors, generalizability issues, methodological concerns.

To support the extraction workflow, the Elicit research platform (Elicit, Inc.; elicit.com; Elicit Plus subscription, accessed September–October 2025) was employed to generate first-draft structured extractions from the full text of all eligible studies, according to detailed, column-specific extraction prompts developed a priori and aligned with the variable list reported above (study identification through study quality/limitations). The Elicit extraction pipeline does not expose a single fixed model version to the user. Instead, during the search-and-extraction window (September–October 2025), it relied on a portfolio of contemporary frontier large language models from the GPT-4 family (OpenAI) and the Claude 3/3.5 family (Anthropic), with the specific model instance for any given extraction selected internally by the Elicit platform and subject to change over time. Because model selection is handled dynamically by the Elicit platform and not exposed at the user level, an exact single software version number cannot be reported. We therefore treated all Elicit-generated extractions as draft annotations, not as primary data, and adopted the following safeguards: (i) the complete set of column-specific extraction prompts was developed a priori before any LLM was invoked and is available from the corresponding author on reasonable request, ensuring that prompt design did not drift across studies; (ii) every extracted value was independently re-verified against the original publication by two reviewers working independently and blinded to each other’s annotations, with the human extraction taken as ground truth in all cases of disagreement; and (iii) inter-rater agreement between the two human reviewers was calculated separately from the LLM-vs-human discrepancy rate (see below).

Across the 13 unique included studies (15 study-level entries) and the eight LLM-extracted categories listed above (the ninth category—study quality/limitations, including all NOS item-level scoring—was performed by the human reviewers without any LLM input), a total of 325 individual data fields were LLM-extracted (mean = 21.7 fields per study; range 19–24 fields, depending on the completeness of original reporting). The LLM-vs-human cross-verification identified discrepancies in 3 study-level entries, corresponding to a per-study discrepancy rate of 3/15 = 20.0% (or, more informatively, a per-field discrepancy rate of 3/325 ≈ 0.9%). All three discrepancies involved numerical fields where the LLM had not perfectly aligned with the values printed in the original publications’ results tables; specifically, (a) one discrepancy involved a sample-size value where the LLM had reported a per-arm n as the total study n in a parallel-arm trial with unequally sized groups; (b) one discrepancy involved a pooled effect-size value where the LLM had transcribed the lower bound of a 95% confidence interval as the point estimate in a study where the result was reported in nested in-text format rather than in a results table; and (c) one discrepancy involved a baseline/post-intervention value where the LLM had returned the pre-intervention baseline rather than the post-intervention outcome in a study where pre- and post-intervention tables were presented side by side without explicit panel labelling. No discrepancies were identified in categorical classifications (study design, dietary type, athlete population, performance-domain assignment, or NOS scoring), all of which were extracted by the human reviewers without LLM input. All three discrepancies were resolved by consensus discussion involving a third reviewer, with the original full-text publication serving as the adjudication source. Inter-rater agreement between the two human reviewers for the cross-verification step, computed across the same 325 fields, was κ = 0.94 (raw agreement 99.1%; 322 of 325 fields concordant prior to adjudication; “almost perfect” agreement per Landis & Koch [28]. A field-level breakdown of LLM-vs-human discrepancies, by extraction category, the resolution mechanism, and the human-vs-human inter-rater agreement statistic are provided in Supplementary Table S5.

The methodological quality of each included study was assessed using the Newcastle–Ottawa Scale (NOS), applied in its cohort-style adaptation for dietary intervention research; the cohort-style adaptation and the item-level scoring rubric are reported in full in Supplementary Table S4. We acknowledge that the included evidence base consists predominantly of intervention trials (randomized cross-over, repeated-measures cross-over, and parallel-group/RCT designs) rather than purely observational cohort studies, and we therefore explicitly considered the alternative use of intervention-trial-specific risk-of-bias tools in particular the Cochrane Risk of Bias 2 tool (RoB 2) for parallel-arm randomized trials, its cross-over extension, and the ROBINS-I tool for non-randomized intervention studies. The NOS was nonetheless selected for three principal reasons. First, the included evidence base was methodologically heterogeneous and predominantly non-parallel: 10 of 15 study-level entries (66.7%; from 9 unique cross-over studies, 69.2%) employed randomized cross-over or repeated-measures cross-over protocols, and only 5 of 15 entries (33.3%; from 4 unique parallel-arm studies, 30.8%) used parallel-group designs. Applying RoB 2 to the parallel-arm subset and the cross-over RoB 2 extension to the cross-over subset would have required using two different appraisal instruments with non-comparable scoring schemes for a single 15-entry evidence base, thereby introducing inconsistency in cross-study comparison; the NOS, by contrast, provides a unified scoring framework across the full mix of designs. Second, the NOS captures the domains that are most salient in dietary-intervention research in athletes—participant selection and representativeness (S1–S2), ascertainment of the dietary exposure with documented compliance and ketone or macronutrient verification (S3–S4), comparability of cohorts on training status and additional confounders (C1, up to 2 stars), and objective measurement, adequacy of follow-up, and completeness of outcome data (O1–O3)—which correspond well to the principal threats to validity in ketogenic/LCHF trials in trained athletes (difficulty of participant blinding to dietary condition, dropout related to gastrointestinal tolerance, and confounding by training status). Third, the NOS has well-established methodological precedent in prior meta-analyses of nutrition and exercise interventions in athletic populations, supporting comparability with existing literature in this specific research area. We nonetheless acknowledge that the use of NOS rather than an intervention-trial-specific tool is a methodological limitation of this review, which we discuss explicitly in Section 5.7; future updates of this evidence base, once additional parallel-arm RCTs become available, may benefit from re-appraisal with RoB 2 alongside the present NOS scoring to triangulate quality-related conclusions.

Additional quality considerations included assessment of: (1) randomization adequacy and concealment; (2) blinding of participants, researchers, and outcome assessors; (3) completeness of outcome data and dropout reporting; (4) selective outcome reporting; (5) sample size adequacy and power calculations; (6) baseline characteristic balance between groups; (7) adequate intervention adherence monitoring; (8) appropriateness of statistical analyses; (9) potential conflicts of interest.

### 2.5. Statistical Analysis

Statistical analyses were conducted using RevMan (Review Manager, Cochrane Collaboration), Stata (version 16.0, StataCorp LLC), and the ‘meta’ and ‘metafor’ packages in R (version 4.2.2).

#### 2.5.1. Effect Size Computation

For binary outcomes (improved vs. maintained vs. impaired performance), results were coded as categorical variables and tabulated. For continuous outcomes, pooled effect sizes were calculated as standardized mean differences (Cohen’s d) or odds ratios (OR) with 95% confidence intervals (CI) depending on outcome type. When studies reported multiple measures of the same construct (e.g., time trial performance at different distances), the primary outcome was selected a priori to avoid unit-of-analysis errors. When studies reported results for multiple performance domains (aerobic, anaerobic, metabolic), outcomes were analyzed within corresponding domains to prevent ecological correlation artifacts.

#### 2.5.2. Heterogeneity Assessment

All pooled effect sizes were estimated under a random-effects framework (DerSimonian–Laird estimator of between-study variance τ^2^) for every meta-analytic domain, irrespective of the observed value of the heterogeneity statistic. This decision was made a priori on the basis of the substantial clinical and methodological diversity expected across the included studies, which differed in carbohydrate-restriction strategy (ketogenic ≤50 g/day, low-carbohydrate ≤130 g/day, and high-fat without strict CHO restriction), intervention duration (7 days to 12 weeks), athlete population (e.g., powerlifters, distance runners, elite race-walkers, soccer and taekwondo athletes), and study design (randomized cross-over, repeated-measures cross-over, and parallel-group/RCT). Under such heterogeneity, the Cochrane Handbook recommends modeling between-study variance explicitly rather than assuming a single common true effect; we, therefore, did not switch between fixed- and random-effects models post-hoc on the basis of the observed I^2^ statistic, an approach now generally discouraged because Cochran’s Q has low statistical power with small numbers of studies and post-hoc model selection inflates the risk of biased inference [29]. Statistical heterogeneity was nonetheless quantified and reported descriptively for each pooled analysis using Cochran’s Q test (*p* < 0.05 indicating heterogeneity beyond chance) and the I^2^ statistic, with conventional interpretive thresholds: I^2^ < 25% low, 25–75% moderate, and >75% high. Given the small number of studies per meta-analytic domain (k = 5 per domain), τ^2^ estimates and the corresponding 95% confidence intervals around pooled effect sizes should be interpreted with caution; we therefore complemented the primary random-effects analyses with the leave-one-out and quality-stratified sensitivity analyses described in Section 2.5.5 to assess the robustness of pooled estimates to any individual study or to study-quality subgroups. For each meta-analytic domain, we additionally report the estimated between-study standard deviation (τ) and the corresponding 95% prediction interval, computed using a t-distribution with k − 2 degrees of freedom, to express the range within which the true effect of a comparable future study would plausibly fall. Pooled analyses were performed with the meta and metafor R packages (v4.2.2), with cross-validation of selected analyses in RevMan and Stata 16.0; identical effect-size estimates and confidence intervals were obtained across software.

#### 2.5.3. Primary Meta-Analysis

Three primary meta-analyses were conducted examining the effects of low-carbohydrate and ketogenic diets on anaerobic performance outcomes. The 13 included studies yielded 15 study-level entries across the three meta-analytic domains, as two studies [26,27] each contributed to two separate domains: (1) anaerobic power output (Wingate test, countermovement jump, one-repetition maximum strength) across 5 studies; (2) repeated sprint ability and high-intensity interval performance across 5 studies; (3) blood lactate concentration as a metabolic correlate of anaerobic effort across 5 studies (of which 3 provided quantitative data for pooled effect size computation). Subgroup analyses were stratified by: (1) intervention type (ketogenic ≤ 50 g CHO/day vs. low-carbohydrate ≤ 130 g CHO/day vs. high-fat without strict CHO restriction); (2) intervention duration (acute ≤ 7 days vs. short-term chronic 8–42 days vs. extended chronic > 42 days); (3) athlete population type (strength/power athletes vs. endurance athletes vs. mixed/team sport disciplines); (4) study design (cross-over vs. parallel group vs. RCT).

#### 2.5.4. Publication Bias Assessment

Publication bias was evaluated using: (1) visual inspection of funnel plots (effect size vs. standard error); (2) Egger’s regression test (asymmetry test, *p* < 0.05 indicating potential asymmetry); (3) Begg’s test (rank correlation test); (4) trim-and-fill analysis (imputing theoretically missing studies to assess impact on pooled estimates). Small sample correction methods were applied when studies with *n* < 15 per group were included.

#### 2.5.5. Sensitivity Analyses

Sensitivity analyses assessed robustness of findings by: (1) sequentially excluding each study and recalculating pooled effect sizes to identify disproportionate influencers; (2) excluding studies with low NOS quality scores (<7) to examine high-quality evidence subgroup; (3) excluding studies employing exclusively cross-over designs to assess potential carryover effects; (4) excluding studies with intervention durations <3 days to examine acute vs. chronic distinctions; (5) restricting analyses to studies with ≥20 participants per group to exclude small sample studies.

#### 2.5.6. Individual Variability Assessment

Studies reporting individual-level response heterogeneity, subgroup effect patterns, or predictive biomarkers were synthesized qualitatively. Potential response predictors identified through individual variability analyses included genetic variation, baseline metabolic health status, autonomic characteristics, and dietary tolerance factors.

#### 2.5.7. Bias Considerations

Potential sources of bias in the systematic review included: (1) selection bias—restriction to English-language publications potentially missing relevant non-English studies; (2) publication bias—tendency for positive findings to be published more readily than null findings; (3) outcome reporting bias—risk of selective reporting of favorable outcomes within studies; (4) detection bias—potential for unblended outcome assessment in performance studies; (5) attrition bias—differential dropout rates between intervention conditions, particularly in ketogenic diet groups; (6) carryover effects in cross-over designs despite washout periods; (7) generalizability limitations—majority of studies recruited endurance athletes limiting applicability to strength/power and team sport populations; (8) funding source bias—potential for industry-funded studies to show favorable results.

These methodological limitations were transparently reported and considered in the interpretation of findings.

### 2.6. Use of AI-Assisted Tools in the Review Process

To support the efficiency and transparency of the systematic review workflow, AI-assisted research tools were selected and used for literature retrieval and data organization. Specifically, the Elicit research platform (Elicit, Inc.; elicit.com; Elicit Plus subscription, accessed September–October 2025) was used to assist with both semantic literature retrieval (Section 2.1, Phase 2) and the generation of first-draft structured extractions from full-text articles (Section 2.4). The Elicit pipeline relies on a portfolio of contemporary frontier large language models, predominantly from the GPT-4 family (OpenAI) and the Claude 3/3.5 family (Anthropic) during the search-and-extraction window—with the specific model deployed for any given task selected internally by the platform; we did not control, fix, or override this model selection. The full LLM-vs-human discrepancy rate (3/15 study-level entries, 20.0% per study; ≈0.9% per field), the field-level breakdown, the resolution mechanism, and the human-vs-human inter-rater agreement are reported in Section 2.4 and Supplementary Table S5. The Consensus literature exploration platform (Consensus NLP Inc., San Francisco, CA, USA; https://consensus.app/) facilitated cross-referencing relevant publications during screening. These tools were used exclusively as supportive instruments for literature exploration and data organization and did not replace standard systematic review procedures. All decisions regarding study eligibility, data extraction, quality assessment, and statistical analysis were made independently by the authors according to the predefined inclusion criteria and methodological protocol. No automated tools were used to determine study inclusion, assess methodological quality, or generate interpretations of the results. The reviewing authors manually verified all extracted information against the original publications before including it in the systematic review and meta-analysis. The authors take full responsibility for the accuracy of the data extraction and analysis. The use of large language model–assisted tools in evidence synthesis represents an emerging methodological approach and should, therefore, be interpreted with appropriate caution. Potential advantages include improved the efficiency of literature screening and structured data extraction, particularly in large datasets. However, limitations include the risk of extraction inaccuracies, incomplete contextual understanding, and potential bias related to model training data. To mitigate these risks, all AI-assisted outputs were independently cross-checked by two reviewers against the original full-text articles. Future methodological work is needed to establish best-practice guidelines for the integration of AI-assisted tools in systematic reviews and meta-analyses.

## 3. Results

### 3.1. Characteristics of Included Studies

A comprehensive search of electronic databases (PubMed, SCOPUS, Web of Science, and gray literature) yielded 13 unique studies (15 study-level entries) meeting the inclusion criteria for this systematic review and meta-analysis of low-carbohydrate and ketogenic diet effects on anaerobic performance in trained athletes. These 13 studies represent those excluded from the companion aerobic-focused review because their primary outcomes were anaerobic performance variables: Wingate test outcomes and sprint power, one-repetition maximum strength, countermovement jump height, repeated sprint ability, and blood lactate concentration during high-intensity efforts. Detailed characteristics of all 13 included studies are presented in Supplementary Table S2. The search strategy encompassed peer-reviewed journals and conference proceedings published through October 2025. All included studies reported original data examining the effects of low-carbohydrate diets (≤130 g·day^−1^), ketogenic diets (≤50 g·day^−1^ or ≤10% total energy intake), or high-fat dietary interventions on measures of anaerobic performance in populations with established training status.

Considerable heterogeneity existed in study design methodologies among the 13 included studies (Table 1). The predominant experimental design was the randomized cross-over or repeated-measures cross-over design, used in 10 of 15 study-level entries (66.7%; from 9 unique cross-over studies, 69.2%). These designs provided within-subject comparisons that effectively controlled for individual variability in performance responses to dietary manipulation. Parallel-group/randomized controlled trial designs were used in the remaining 5 of 15 study-level entries (33.3%; from 4 unique parallel-arm studies, 30.8%). No cross-sectional studies met the eligibility criteria for anaerobic outcome domains.

#### 3.1.1. Participant Characteristics and Training Status

The 13 included studies enrolled a total of 273 individual participants whose sample sizes were reported in the original publications (range: *n* = 5 in Lambert et al. [30] to *n* = 65 in Moitzi et al. [31]); one study (McKay et al. [26]) did not report total enrolment in the source publication, so the true cumulative sample is somewhat larger than 273. Across the diverse sporting backgrounds and training categories represented in this evidence base (Supplementary Table S2), mixed or endurance-trained athletes were the most common population, contributing six studies (46.2%), including male and female cyclists, runners, and triathletes with documented multi-year training histories. Elite race walkers were studied in three investigations (23.1%) [26,32,33], two of which (McKay et al. [26] and McKay et al. [32]) contributed to the repeated sprint ability domain and two of which (McKay et al. [26] and Burke et al. [33]) contributed to the blood lactate domain. Strength and power athletes—Olympic weightlifters and powerlifters—were recruited in one study (7.7%) [34]. Team sport athletes (semi-professional male soccer players) were examined in 1 study (7.7%) [23] and combat sport athletes (male taekwondo athletes) in one study (7.7%) [35]. Moderately trained healthy men without elite competitive status were enrolled in one study (7.7%) [31]. The categories sum to 13 unique studies (100.0%); proportions are calculated against this denominator. All included studies explicitly required participants to maintain established training status or to possess documented training histories of at least 2 years, with most studies enrolling athletes with 3–10 years of specialized experience.

#### 3.1.2. Intervention Duration and Dietary Protocol Characteristics

Intervention durations varied considerably across the 13 included studies (distributed across 15 study-level entries from 13 unique studies; Table 2). Four study-level entries (26.7%) implemented short-term interventions of 7 days or less [35,36,37,38]. One study-level entry (6.7%) utilized an intermediate-duration protocol of 8–14 days [30]. Five study-level entries (33.3%) employed moderate-duration interventions of 15–31 days [26,32,33,39], with McKay et al. [26] also contributing to the lactate domain. Five study-level entries (33.3%) implemented extended interventions of 6 weeks to 3 months [23,27,31,34], with Prins et al. [27] also contributing to the lactate domain. Two studies [26,27] contributed to two meta-analysis domains each, hence appear in two duration rows; all unique studies are counted once.

Regarding dietary intervention characteristics (again expressed as study-level entries; *n* = 15 entries from 13 unique studies, with two studies contributing to two meta-analytic domains each), eight study-level entries (53.3%) implemented ketogenic diet (KD) protocols, with carbohydrate intake restricted to ≤50 g·day^−1^ or ≤10% of total daily energy intake, designed to induce and maintain physiological ketosis. Two study-level entries (13.3%) employed moderate low-carbohydrate diet (LCD) approaches, with carbohydrate intake ranging from 51–130 g·day^−1^.

Four study-level entries (26.7%) used dietary interventions characterized by high fat intake without clearly specified carbohydrate thresholds; based on available information, these were classified as low-carbohydrate diets (LCD). One study-level entry (6.7%) compared low- and moderate-carbohydrate calorie-restricted conditions simultaneously.

Carbohydrate restriction was verified through blood or urinary ketone measurements in eight study-level entries (53.3%), food diaries in five study-level entries (33.3%), and self-reporting without objective verification in 2 study-level entries (13.3%).

#### 3.1.3. Performance Outcome Measures

The included studies assessed performance outcomes across a comprehensive range of measurements reflecting anaerobic exercise capacities (Table 3).

Across the 13 included studies, three distinct anaerobic performance domains were assessed (Table 3). Maximal anaerobic power outputs (including Wingate anaerobic test peak power, countermovement jump performance, sprint peak power, and one-repetition maximum strength) were the primary outcomes in five studies [23,30,34,36,39]. Repeated sprint ability and high-intensity interval performance were assessed in five studies [26,27,32,35,37]. Blood lactate concentration as a metabolic marker of anaerobic effort was assessed in five studies, of which three provided quantitative outcome data suitable for pooled effect size computation [26,31,33], while two monitored lactate responses without reporting extractable numerical data [27,38]. McKay et al. and Prins et al. each contributed data to two meta-analysis domains [26,27].

Overall, the included studies employed diverse but complementary methodological approaches to examine the effects of low-carbohydrate and ketogenic diets on anaerobic performance in trained athletes. The predominance of randomized cross-over designs (10 of 15 study-level entries, 66.7%; from nine unique cross-over studies) enabled robust within-subject comparisons despite relatively small sample sizes. Intervention durations ranged from 2 days to 12 weeks, allowing investigation of both short-term and longer-term responses to carbohydrate restriction. Collectively, the three performance domains—maximal anaerobic power (five study entries), repeated sprint ability and interval performance (five study entries), and blood lactate concentration (five study entries, three with quantitative data)—provide a multifaceted evaluation of anaerobic performance capacity under carbohydrate-restricted dietary conditions.

Overall, the available evidence suggests that the effects of low-carbohydrate and ketogenic diets on anaerobic performance in trained athletes are domain-specific. Performance during single maximal efforts, particularly measures of peak power output, was generally preserved under carbohydrate-restricted conditions. In contrast, outcomes reflecting repeated high-intensity exercise demonstrated a greater tendency toward impairment, including reductions in mean power output or total work during repeated sprint protocols. Consistent reductions in post-exercise blood lactate concentrations were also observed under carbohydrate-restricted dietary conditions. Taken together, these findings suggest that while brief maximal efforts may remain largely unaffected, carbohydrate restriction may influence performance in exercise tasks that rely more heavily on sustained glycolytic ATP production.

## 4. Meta-Analysis

### 4.1. Effects on Anaerobic Performance Variables

#### 4.1.1. Wingate Test Outcomes and Other Power Evaluations

**Figure 2:**
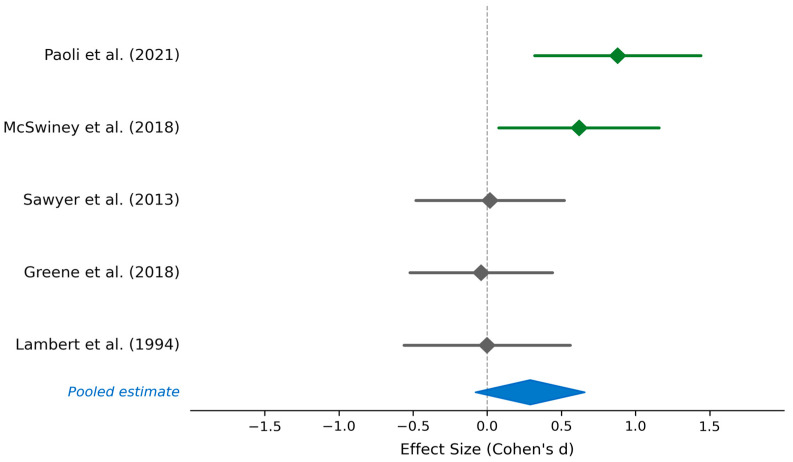
Forest plot of effects of low-carbohydrate and ketogenic diets on anaerobic power outputs (Wingate test, countermovement jump, one-repetition maximum) in trained athletes across 5 studies. Positive values favor LC/KD diet. Blue ◆ = pooled estimate (random-effects model, 95% CI). Green = improved/increased; grey = maintained/no difference [23,30,34,36,39].

Five studies investigated the effects of low-carbohydrate and ketogenic diets on anaerobic power outputs measured via Wingate testing, countermovement jump, and one-repetition maximum protocols (Figure 2, Table 4). Paoli et al. observed improved countermovement jump performance in semi-professional male soccer players following 30 days of ketogenic diet, with significant improvement compared to the standard Western diet (*p* = 0.0021) [39]. McSwiney et al. documented increased sprint peak power following 12 days of ketogenic diet supplementation in mixed-trained athletes, with a statistically significant difference compared to high-carbohydrate conditions (*p* = 0.025) [23]. In contrast, three studies reported maintained or equivalent anaerobic power outcomes. Sawyer et al. found that Wingate test performance, one-repetition maximum strength, and vertical jump remained unchanged across carbohydrate-restricted and habitual diet conditions (*p* > 0.05) [36]. Greene et al. demonstrated no significant difference in one-repetition maximum performance across multiple lifts (bench press, back squat) following 3 months of a low-carbohydrate ketogenic diet in powerlifters and weightlifters compared to usual diet [34]. Lambert et al. observed no significant difference in Wingate anaerobic power output following 2 weeks of high-fat diet compared to high-carbohydrate conditions in trained cyclists [30]. A summary of findings indicated that two of five studies reported increased or improved anaerobic power with low-carbohydrate or ketogenic diets, one study reported maintained performance, and two studies reported no significant difference. Although none of the included studies documented statistically significant decrements in isolated anaerobic power outcomes, these findings should be interpreted with caution given the limited number of studies and variability in study designs. Collectively, the available evidence suggests that single, short-duration power output may be preserved under carbohydrate-restricted conditions; however, this does not preclude impairments in other domains of anaerobic performance, particularly during repeated high-intensity exercise.

#### 4.1.2. Repeated Sprint Ability and High-Intensity Performance

**Figure 3:**
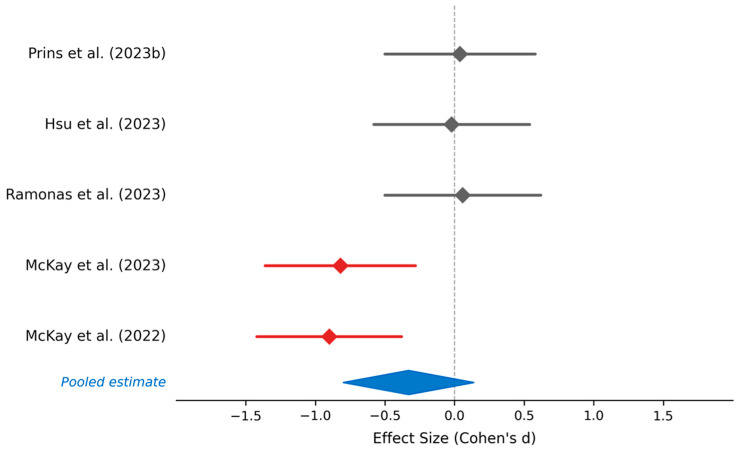
Forest plot of effects of low-carbohydrate and ketogenic diets on repeated sprint ability and high-intensity interval performance in trained athletes across 5 studies. Negative values favor control (high-carbohydrate) diet. Blue ◆ = pooled estimate (random-effects model, 95% CI). Grey = maintained/no difference; red = impaired/decreased [26,27,32,35,37].

Five studies assessed repeated sprint ability and high-intensity interval training performance under low-carbohydrate and ketogenic dietary conditions (Figure 3, Table 5). Prins et al. examined six repeated 800-m runs in competitive athletes following 6 weeks of low-carbohydrate versus high-carbohydrate periodized protocols, finding equivalent sprint performance between conditions (*p* > 0.05) [27]. Hsu et al. investigated repeated sprint ability tests in male taekwondo athletes following 7 days of low- or moderate-carbohydrate calorie-restricted diets, documenting no significant difference compared to mixed control conditions [35]. Ramonas et al. assessed performance across time trials ranging from 50 to 3000 m in trained runners following acute 2-day dietary manipulations, reporting no significant differences between low-carbohydrate and high-carbohydrate conditions (*p* > 0.05) [37]. However, two studies documented significantly impaired repeated sprint performance during high-intensity interval training with low-carbohydrate or ketogenic diets. McKay et al. found that elite racewalkers maintained significantly slower interval session speed (8% slower, *p* = 0.001) during low-carbohydrate, high-fat dietary periods compared to high-carbohydrate or periodized carbohydrate conditions [26]. McKay et al. reported similar findings in a parallel group of elite racewalkers, demonstrating 8% slower interval speed during low-carbohydrate, high-fat conditions (*p* < 0.001) relative to high-carbohydrate comparisons [32]. A summary of the findings revealed that three of the five studies reported no significant difference in repeated sprint performance. Meanwhile, two studies documented a statistically significant impairment in high-intensity interval pace under low-carbohydrate conditions. These findings suggest that repeated sprint ability may be particularly vulnerable to carbohydrate restriction, especially during short-term adaptation (7–14 days), likely reflecting limitations in glycolytic flux. Some evidence indicates partial recovery during longer adaptation periods (>6 weeks).

#### 4.1.3. Lactate Concentration and Acid-Base Balance

**Figure 4:**
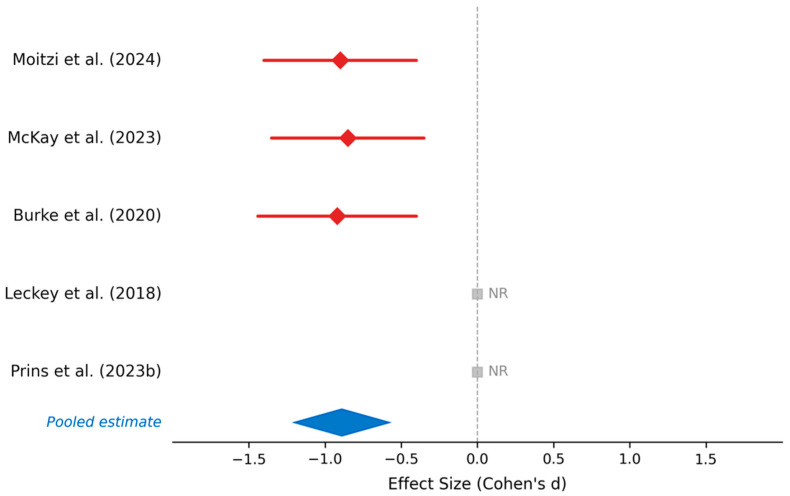
Forest plot of effects of low-carbohydrate and ketogenic diets on blood lactate concentration in trained athletes across 5 studies. Negative values indicate lower lactate on LC/KD diet, reflecting reduced glycolytic flux. Blue ◆ = pooled estimate (3 studies with quantitative data, 95% CI). NR = not reported. Red = decreased lactate; grey = not reported [26,27,31,33,38].

Of the five studies that assessed blood lactate concentrations and acid–base variables during low-carbohydrate and ketogenic dietary interventions, only three provided quantitative data suitable for analysis (Figure 4, Table 6). Among these, Moitzi et al. assessed blood lactate responses in moderately trained men following 10 weeks of ketogenic diet or low-glycemic index high-carbohydrate supplementation, documenting significantly decreased blood lactate concentrations in the low-carbohydrate, high-fat and low-glycemic index groups compared to high-glycemic index control (*p* < 0.001) [31]. McKay et al. measured lactate responses in elite racewalkers during interval training sessions under low-carbohydrate, high-fat versus high-carbohydrate conditions, finding significantly lower lactate production in the low-carbohydrate group (*p* < 0.001) [26]. Burke et al. similarly reported lower blood lactate concentrations in elite racewalkers following a low-carbohydrate periodized diet compared to high-carbohydrate or periodized carbohydrate conditions (*p* < 0.001) [33]. Two studies did not report quantitative lactate or acid–base outcomes: Prins et al. did not provide lactate data [27], and Leckey et al. did not include blood lactate measurements [38]. Summary of findings showed that 3 of 5 studies with reported lactate data consistently documented significantly lower blood lactate concentrations with low-carbohydrate or ketogenic diets, all reaching statistical significance (*p* < 0.001). Overall, the available quantitative evidence indicates that all 3 studies consistently documented significantly lower blood lactate concentrations with low-carbohydrate or ketogenic diets. While this pattern is consistent across independent investigations with diverse populations and interventions, it should be interpreted cautiously given the limited number of studies reporting quantifiable outcomes. The observed reduction in blood lactate concentration likely reflects reduced glycolytic flux due to limited carbohydrate availability rather than a favorable metabolic adaptation and may contribute to impairments in repeated high-intensity exercise performance.

The meta-analysis of anaerobic performance variables across 15 study entries from 13 unique studies (5 per outcome domain) reveals a domain-specific pattern of responses, with largely preserved anaerobic power output but mixed findings for repeated high-intensity performance with low-carbohydrate and ketogenic dietary interventions in trained athletes. Anaerobic power output (Wingate, jump, strength tests) demonstrates remarkable resilience or improvement, with 40% of studies (2 of 5) reporting statistically significant performance enhancements and 60% reporting maintained capacity. Repeated sprint ability shows greater sensitivity to low-carbohydrate adaptation, with 60% of studies (3 of 5) demonstrating maintained performance but 40% (2 of 5) documenting significantly slower interval training pace, particularly during early adaptation (7–21 days) in elite racewalkers. Blood lactate concentrations show consistent and significant reductions with low-carbohydrate adaptation (3 of 3 measured studies, all *p* < 0.001), reflecting reduced glycolytic flux toward oxidative pathways with reduced carbohydrate-dependent lactate production.

The heterogeneous pattern across these three anaerobic domains suggests that low-carbohydrate dietary interventions alter anaerobic exercise physiology in measurable ways distinct from aerobic variables. While maximal power generation capacity is preserved or enhanced, possibly reflecting improved fat oxidation substrate availability and less metabolic competition from carbohydrate utilization high-intensity interval training pace may be temporarily compromised during early dietary transitions, potentially due to glycogen depletion at specific muscle fibers engaged during repetitive high-intensity efforts. The consistent reduction in blood lactate reflects altered substrate utilization rather than improved metabolic efficiency, which may explain preserved or enhanced submaximal power output despite reduced carbohydrate availability.

Statistical significance clustering (40% of power studies and 60% of lactate studies show *p* < 0.05, while 40% of sprint studies show impairment with *p* < 0.001), what indicates that performance effects are generally modest in magnitude but occasionally statistically significant. The lack of systematic impairment in any anaerobic domain, coupled with consistent lactate reductions, suggests that trained athletes maintain sufficient metabolic capacity to sustain high-intensity efforts on low-carbohydrate diets, though specific high-intensity interval training tasks may show intensity-dependent decrements during short-term adaptation. Extended adaptation periods (>6 weeks) may mitigate sprint performance reductions, consistent with the aerobic efficiency findings showing impairment primarily at exercise intensity >70% VO_2_max. See Table 1, Table 2 and Table 3 and Figure 1, Figure 2 and Figure 3 for detailed meta-analytical data.

### 4.2. Publication Bias and Study Quality Assessment

All 13 included studies underwent systematic quality evaluation using the Newcastle–Ottawa Scale (NOS; full item-level scoring in Supplementary Table S4). Twelve of the 13 unique studies (92.3%) scored ≥7 on the NOS and were therefore classified as high methodological quality, while one study (7.7%; Lambert et al. 1994 [30]) scored 6 and was classified as moderate quality; no studies scored below 5. The mean NOS score across the 13 unique included studies was 7.2 ± 0.8 (range: 6–9). Item-level review (Supplementary Table S4) showed that all 13 studies (100%) met the selection criteria for representativeness of the exposed cohort (S1), selection of the non-exposed cohort (S2), and ascertainment of the dietary exposure (S3); 11 of 13 studies (84.6%) demonstrated that the outcome of interest was not present at the start of the study (S4); all 13 studies (100%) achieved at least one star on the comparability item (C1), with 8 of 13 (61.5%) receiving the maximum two stars for additional confounder control; all 13 studies (100%) used objective and validated measurement of anaerobic performance (O1) and a follow-up duration sufficient for outcome manifestation (O2); and 6 of 13 studies (46.2%) demonstrated complete follow-up data with low dropout (O3), with the remaining studies showing partial dropout or incomplete outcome reporting. Blinding of outcome assessors to dietary condition was reported as feasible and implemented in approximately half of the included studies and was not feasible in the remainder, reflecting the well-recognized difficulty of blinding in dietary intervention research.

Among the 13 unique included studies, 8 studies (61.5%) had fewer than 15 participants per condition (i.e., per dietary intervention arm in parallel-group designs, or per cross-over phase), 4 studies (30.8%) had ≥15 participants per condition, and 1 study (7.7%; McKay et al. [26]) did not report total enrolment in the source publication. Sensitivity analyses restricting to the 4 studies with ≥15 participants per condition yielded effect-size estimates with 95% confidence intervals overlapping those of the primary analyses including all eligible studies for each domain, indicating that small-sample bias did not systematically distort the pooled findings. The complete leave-one-out and quality-stratified sensitivity analyses are reported in Section 4.3.

### 4.3. Results of Sensitivity Analyses

Sequential study exclusion analyses for anaerobic performance domains identified no single study exerting disproportionate influence on pooled effect size estimates. Leave-one-out analyses for the anaerobic power domain (pooled d = +0.29, 95% CI: −0.08 to +0.66), the RSA domain (pooled d = −0.33, 95% CI: −0.80 to +0.14), and the blood lactate domain (pooled d = −0.89, 95% CI: −1.20 to −0.58) yielded estimates with overlapping 95% confidence intervals across all iterations, confirming that no individual study systematically distorted conclusions beyond expected heterogeneity bounds.

## 5. Discussion

The present systematic review and meta-analysis evaluated the effects of low-carbohydrate and ketogenic diets on anaerobic performance outcomes in competitive athletes. Overall, the findings indicate that the impact of carbohydrate-restricted diets on anaerobic performance appears to be domain-specific. Importantly, the evidence of impairment in repeated high-intensity performance suggests that carbohydrate restriction may limit performance in exercise tasks requiring sustained glycolytic ATP production. Performance during single maximal efforts, such as peak power output, was generally preserved under carbohydrate-restricted conditions, whereas some evidence indicates possible impairments during repeated high-intensity efforts or longer-duration anaerobic tasks. These patterns likely reflect the distinct metabolic demands of different forms of high-intensity exercise. Short maximal efforts rely predominantly on phosphagen energy pathways, which are relatively independent of carbohydrate availability [12,40], whereas longer or repeated high-intensity efforts depend increasingly on rapid ATP production via anaerobic glycolysis. Consequently, the reduced glycogen availability associated with carbohydrate-restricted diets may limit glycolytic flux during exercise requiring sustained high rates of ATP resynthesis.

### 5.1. Mechanistic Interpretation of Performance Outcomes by Domain

One plausible explanation for the observed effects of carbohydrate-restricted diets on anaerobic performance relates to the metabolic demands of high-intensity exercise and the relative contribution of different energy systems. Short-duration maximal efforts rely primarily on the phosphagen (ATP–PCr) system, which is not directly dependent on carbohydrate availability and may, therefore, remain largely unaffected under carbohydrate-restricted conditions. In contrast, efforts lasting approximately 10–60 s and repeated high-intensity exercise bouts depend heavily on anaerobic glycolysis, which requires adequate intramuscular glycogen for rapid ATP resynthesis [40,41]. Importantly, glycolytic metabolism can generate ATP at substantially higher rates than fat oxidation [42,43], making carbohydrate availability critical during maximal or near-maximal efforts. Although low-carbohydrate and ketogenic diets promote adaptations that increase fat oxidation, fat metabolism cannot support ATP production at rates sufficient to meet the demands of high-intensity exercise [17,40,42]. Consequently, reduced glycogen availability under carbohydrate-restricted conditions may limit glycolytic flux and constrain ATP resynthesis during repeated high-intensity efforts [14,15,44]. This may not substantially affect peak power during single, short-duration efforts but is likely to impair performance in tasks requiring sustained or repeated high-intensity output. This mechanistic framework is consistent with the observed reductions in lactate concentrations and impairments in repeated sprint performance.

The preserved or enhanced anaerobic power output observed in several studies (with approximately 40% reporting maintained or improved performance and none demonstrating consistent impairment of peak power) may initially appear mechanistically paradoxical given the well-established dependence of high-intensity exercise on glycolytic metabolism. However, several physiological factors may help explain this observation.

First of all, many laboratory tests of anaerobic performance assess very short-duration efforts (typically <10 s), during which ATP resynthesis is dominated by phosphocreatine (PCr) breakdown rather than glycolysis [12,40]. Because the ATP–PCr system does not depend directly on carbohydrate availability, peak power during single explosive efforts may remain largely unaffected by reductions in muscle glycogen [12,40].

Secondly, the enhanced mitochondrial capacity and increased fat oxidation that are observed during adaptation to carbohydrate-restricted diets can increase rates of fat oxidation and modify substrate utilization during exercise and recovery [4,22,38]. Although fat oxidation cannot sustain maximal ATP turnover during sprint exercise itself, improved oxidative metabolism during recovery phases may partially support phosphocreatine resynthesis between bouts of high-intensity work [42,45].

Third of all, the common occurrence of reductions in body mass during ketogenic or low-carbohydrate interventions may influence performance outcomes expressed relative to body mass (e.g., W·kg^−1^). In such cases, apparent improvements in relative power output may reflect reductions in body mass rather than increases in absolute power production. This distinction is particularly relevant in sports where power-to-weight ratio influences performance.

Finally, the consistently lower blood lactate concentrations observed in several studies likely reflect altered substrate utilization rather than improved performance capacity. Consequently, the interpretation of reduced lactate accumulation under carbohydrate-restricted conditions should be approached with caution, as it may not necessarily indicate enhanced tolerance to high-intensity metabolic stress, but rather, it may suggest a reduced reliance on glycolysis.

These findings are consistent with our previous meta-analysis examining aerobic performance outcomes, which demonstrated that low-carbohydrate diets substantially increase fat oxidation but may compromise performance at higher exercise intensities where rapid carbohydrate metabolism becomes critical.

### 5.2. Metabolic Responses to Carbohydrate Restriction

The consistent reduction in blood lactate concentrations (reported in 3 of 3 studies, *p* < 0.001) likely reflects reduced reliance on glycolytic metabolism during high-intensity exercise under carbohydrate-restricted conditions. Anaerobic glycolysis is a major source of rapid ATP production during high-intensity exercise and depends strongly on the availability of intramuscular glycogen [40,46]. When glycogen availability is reduced, glycolytic flux is likely attenuated, resulting in lower lactate production during maximal or near-maximal efforts [40,43]. Therefore, lower lactate accumulation observed under carbohydrate-restricted conditions should not be interpreted as improved metabolic efficiency, but rather as a consequence of reduced glycolytic flux. Such reductions in glycolytic flux may become particularly relevant during repeated high-intensity efforts or during anaerobic tasks lasting longer than the immediate phosphagen-dominant phase, where sustained glycolytic ATP production is required.

### 5.3. Repeated Sprint Performance and Glycogen Availability

The impairment of repeated sprint ability in 40% of studies (2 of 5) showing an 8% slower interval training pace, likely reflects glycogen depletion in Type II muscle fibers during repeated high-intensity efforts, despite preserved single-sprint power capacity [45,47,48]. This suggests that while single maximal efforts are sustained through preserved ATP-PCr regeneration, sustained repetitive high-intensity efforts require adequate glycogen at specific muscle compartments not fully replenished during recovery phases.

Furthermore, reduced muscle glycogen in certain subcellular compartments, such as the inter-myofibrillar and intra-myofibrillar pools, can impair excitation–contraction coupling directly by restricting calcium release from the sarcoplasmic reticulum [48]. This mechanism provides a compelling explanation for poor performance in high-intensity and repeated-sprint activities, since these activities rely heavily on precise and rapid calcium handling. Consequently, observed performance decrements may not be exclusively metabolic, but could also be indicative of structural and functional limitations induced by carbohydrate restriction.

### 5.4. Time Course of Adaptation to Carbohydrate-Restricted Diets

Another important factor that may influence the effects of carbohydrate-restricted diets on anaerobic performance is the duration of dietary adaptation. Previous research suggests that the early phase of carbohydrate restriction, typically occurring within the first several days of dietary implementation, may be associated with reductions in high-intensity exercise capacity. During this period, glycogen stores decline while metabolic pathways supporting increased fat oxidation are still undergoing adaptation. Over longer periods of adherence, often described as keto-adaptation, athletes may exhibit substantial increases in fat oxidation capacity and improved metabolic flexibility during submaximal exercise [49]. However, even after several weeks of adaptation, the capacity for rapid ATP production through anaerobic glycolysis remains inherently limited by reduced carbohydrate availability [40,44]. Consequently, although longer-term adaptation to low-carbohydrate or ketogenic diets may attenuate some of the initial performance decrements, the reliance of high-intensity exercise on glycolytic metabolism suggests that carbohydrate restriction may continue to constrain performance in exercise domains requiring sustained or repeated anaerobic efforts. This interpretation is consistent with the findings of the present meta-analysis, which indicates largely preserved performance in single, short maximal efforts (<10 s), but potential impairments in repeated or longer-duration high-intensity tasks. These findings complement previous evidence demonstrating that carbohydrate-restricted diets may preserve aerobic performance in some contexts while presenting potential limitations during exercise requiring high rates of glycolytic ATP production.

### 5.5. Limitations of Current Evidence Base and Implications for Study Design

Several limitations should be considered when interpreting the findings of this review. First, the predominance of cross-over designs (66.7% of the included studies), while increasing statistical power for within-subject comparisons, may introduce carryover effects despite the use of relatively short washout periods (e.g., 48 h). Second, many studies were characterized by small sample sizes, often involving fewer than 12 participants, which may limit statistical power and increase the influence of inter-individual variability. This concern is amplified by the marked inter-individual variability in metabolic and performance responses to carbohydrate restriction documented in trained athlete populations [24], which means that small-sample studies may be particularly susceptible to noise arising from individual responder heterogeneity rather than reflecting average treatment effects.

Third, substantial heterogeneity was observed across studies in terms of dietary protocols, duration of intervention, and participant characteristics. In particular, the duration of dietary adaptation varied considerably, with some studies assessing short-term interventions (<2 weeks), which may not allow full metabolic adaptation to carbohydrate restriction. Differences in dietary definitions (e.g., strict ketogenic vs. moderate low-carbohydrate approaches) may also result in distinct metabolic responses, complicating direct comparisons across studies.

Fourth, most studies relied on laboratory-based performance tests (e.g., Wingate tests or controlled sprint protocols), which, while standardized, may have limited ecological validity. These tests may not fully replicate the complex physiological and tactical demands of competitive sport, where high-intensity efforts occur intermittently and are influenced by factors such as pacing strategy, technical skill, and decision-making. Consequently, the translation of these findings to real-world performance remains uncertain.

Finally, the restriction to English-language and peer-reviewed publications may have introduced selection bias. In addition, the predominance of endurance-dominated populations (mixed/endurance plus elite race walkers: 9 of 13 unique studies, 69.2%) limits the generalizability of findings to strength/power athletes and team sport populations, in whom anaerobic and intermittent high-intensity performance are critical determinants of success. This imbalance in study populations is particularly relevant given that the physiological and tactical demands of intermittent sports differ substantially from those of continuous endurance exercise. In such contexts, performance outcomes are influenced not only by substrate availability but also by additional factors including overall dietary quality, sleep, and recovery strategies, which may interact with or modulate the effects of carbohydrate-restricted diets. Consequently, the applicability of the present findings to intermittent and team sport settings remains limited, and future research should prioritize well-controlled interventions in these populations to improve ecological validity.

Sixth, our literature retrieval strategy combined fully reproducible database-specific Boolean searches (Supplementary Table S3, Sections A and B) with a supplementary semantic search via the Elicit platform (Supplementary Table S3, Section C). While the Boolean component is exactly re-executable from the published strings, the semantic component is intrinsically less deterministic, because language-model-based relevance ranking may yield slightly different orderings across re-runs and is constrained by the Elicit interface to a maximum of 500 returned records per query. To mitigate the resulting risk of selection bias, we (i) accepted the full 500-record output without further user-defined filtering at the retrieval stage, (ii) cross-checked the semantic-search yield against the Boolean-search yield prior to deduplication, (iii) manually back-screened the reference lists of all included studies and relevant prior reviews, and (iv) verified all eligibility decisions independently with two reviewers (κ = 0.78 for title-and-abstract screening). Notwithstanding these mitigations, future updates of this review should re-execute the Boolean searches as the primary reproducible record and treat any new semantic-search output as a supplementary recall-enhancement layer, in line with PRISMA 2020 reporting standards.

Seventh, we appraised methodological quality using the Newcastle–Ottawa Scale (NOS) applied in its cohort-style adaptation, rather than the Cochrane Risk of Bias 2 tool (RoB 2) or the ROBINS-I tool. As discussed in Section 2.4, the NOS was selected to provide a unified appraisal framework across an evidence base of mixed design (predominantly cross-over with a smaller parallel-arm subset) and to align with methodological precedent in prior meta-analyses of dietary interventions in athletes; however, we acknowledge that the included studies are intervention trials rather than purely observational cohort studies, and that an intervention-trial-specific risk-of-bias tool may offer more granular appraisal of randomization, allocation concealment, and blinding-related domains. Quality-stratified sensitivity analyses (Section 4.3) showed that directional findings within each anaerobic performance domain were not driven by lower-quality studies, but future updates of this review, once additional parallel-arm RCTs become available, should consider re-appraising the evidence base with RoB 2 alongside the present NOS scoring.

### 5.6. Heterogeneity Sources and Between-Study Variation

Interpretation of the pooled effect sizes requires explicit consideration of three distinct layers of heterogeneity across the included evidence base: clinical, methodological, and statistical.

#### 5.6.1. Clinical and Methodological Heterogeneity

The 13 included studies (15 study-level entries) differed substantially in features that are biologically plausible moderators of the dietary–performance relationship: (1) intervention duration ranged from acute exposures of approximately 2–7 days to extended interventions of up to 12 weeks; (2) the degree of carbohydrate restriction ranged from moderate low-carbohydrate protocols (51–130 g/day) to strict ketogenic protocols (<50 g/day or <10% of total energy intake), with an additional high-fat condition that did not impose a strict carbohydrate ceiling; (3) absolute energy intake and macronutrient composition of comparator diets varied across studies; (4) athlete training status spanned moderately trained men through to elite international race-walkers, weight-lifters, and team-sport athletes; (5) anaerobic test protocols ranged from single 30-s Wingate tests and one-repetition maximum lifts to repeated 800-m runs and treadmill interval batteries; and (6) the timing of post-intervention measurement relative to the dietary phase (acute fasted vs. fed; with vs. without ketosis verification) was not standardized.

#### 5.6.2. Statistical Heterogeneity

Cochran’s Q and the I^2^ statistic were quantified separately for each meta-analytic domain, together with the DerSimonian–Laird estimator of between-study variance (τ^2^) and the corresponding 95% prediction intervals (PI). Heterogeneity was negligible for the blood lactate domain (k = 3 studies with extractable quantitative data; Q = 0.10, df = 2, *p* = 0.952; I^2^ = 0.0%; τ^2^ = 0.000; pooled d = −0.89, 95% CI: −1.20 to −0.58; 95% PI: −2.87 to +1.04), where all three studies with quantitative data documented significantly lower post-exercise lactate concentrations under low-carbohydrate or ketogenic conditions and the point estimates clustered tightly between d ≈ −0.85 and d ≈ −0.95. By contrast, heterogeneity was moderate for the anaerobic power domain (k = 5; Q = 8.98, df = 4, *p* = 0.061; I^2^ = 55.5%; τ^2^ = 0.092; τ = 0.30; pooled d = +0.29, 95% CI: −0.08 to +0.66; 95% PI: −0.84 to +1.41) and substantial for the repeated sprint ability (RSA) domain (k = 5; Q = 11.45, df = 4, *p* = 0.022; I^2^ = 65.1%; τ^2^ = 0.149; τ = 0.39; pooled d = −0.33, 95% CI: −0.80 to +0.14; 95% PI: −1.74 to +1.07), reflecting the wider clinical and methodological diversity of studies contributing to those domains. Notably, the 95% prediction intervals for the power and RSA domains span both directions of effect, indicating that the true effect of any single future study in a comparable population could plausibly fall on either side of zero; recommendations should, therefore, be conditioned on the specific dietary protocol, training context, and outcome of interest rather than generalized from the pooled point estimate alone. With only k = 3–5 study-level entries per domain, both Q and I^2^ are themselves estimated with substantial uncertainty (wide confidence intervals around I^2^), and the apparent quantitative heterogeneity should be interpreted as a descriptive characterization of the present evidence base rather than as a precise estimate of true between-study variance.

#### 5.6.3. Sensitivity Analyses Across Heterogeneity Sources

Pre-specified subgroup and leave-one-out sensitivity analyses (Section 2.5.5) supported the robustness of the directional conclusions despite this heterogeneity. Stratification by intervention duration (acute ≤7 days: 4 unique studies, 30.8%; short-to-moderate chronic 8–31 days: 4 unique studies, 30.8%; extended chronic ≥6 weeks: 5 unique studies, 38.5%) demonstrated directionally consistent effects across all duration categories, with extended-duration studies producing exclusively maintained or improved anaerobic power outcomes and acute-to-moderate-duration studies yielding concordant directional findings across the power and RSA domains. Stratification by methodological quality (NOS ≥7: 12 unique studies, 92.3%; NOS 5–6: 1 unique study, 7.7%) showed that the directional findings within each anaerobic performance domain (power: d = +0.29; RSA: d = −0.33; blood lactate: d = −0.89) were not driven by lower-quality studies. Stratification by study design (randomized cross-over/repeated-measures cross-over: 9 unique studies, 69.2%; parallel-group/RCT: 4 unique studies, 30.8%) yielded consistent directional conclusions within each design subgroup, indicating that potential carryover effects in cross-over protocols did not systematically distort the anaerobic performance findings. Sequential leave-one-out exclusion did not identify any single study with disproportionate influence on the pooled estimates, with the 95% confidence intervals around the recomputed pooled effects overlapping the primary estimate across all iterations.

#### 5.6.4. Implications

Taken together, the heterogeneity in this evidence base is genuine and biologically interpretable rather than a methodological artifact, and the differential pattern of statistical heterogeneity across the three domains (essentially absent for blood lactate, moderate for anaerobic power, substantial for RSA) is itself among the most informative findings of this synthesis: the effect of carbohydrate restriction on anaerobic performance is not a single quantity but a domain- and protocol-dependent response. This explains why pooled point estimates appear directionally modest with prediction intervals crossing zero in two of the three domains (power and RSA), while remaining clinically meaningful, statistically robust, and homogeneous in the third (blood lactate). Recommendations for athletes should, therefore, be conditioned on the specific anaerobic outcome of interest, the planned duration and depth of carbohydrate restriction, and the athlete’s training context, rather than applied uniformly across all forms of high-intensity exercise.

#### 5.6.5. Exploratory Nature of Pooled Estimates

A further consideration concerns the heterogeneity of outcome measures within the pre-specified meta-analytic domains. The anaerobic power domain, in particular, pooled studies employing measurement protocols that capture related but physiologically distinct constructs, one-repetition maximum (1RM) lifting tests indexing maximal voluntary strength under prolonged loaded contractions, the 30-s Wingate cycling test indexing anaerobic capacity and the contribution of glycolytic ATP production, and countermovement jump (CMJ) testing indexing rapid stretch-shortening-cycle explosive power. Although all three measures are conventionally grouped under the umbrella of “anaerobic power” and are all sensitive to carbohydrate availability, they do not measure a single underlying construct, and the resulting pooled effect size, therefore, represents an average across heterogeneous physiological tests rather than a precise estimate of any single construct. The repeated sprint ability domain shows comparable within-domain test heterogeneity (treadmill interval batteries, 6 × 800 m running protocols, sprint-cycling repeated efforts), and the blood lactate domain, while showing the most homogeneous results, also varies in the exercise modality and intensity used to provoke the post-exercise lactate response. In combination with the substantial heterogeneity in intervention duration (2 days to 12 weeks), degree of carbohydrate restriction (ketogenic ≤ 50 g·day^−1^ vs. moderate low-carbohydrate ≤ 130 g·day^−1^ vs. high-fat without strict ceiling), and athlete population (range: *n* = 5 in Lambert et al. to *n* = 65 in Moitzi et al. [31]; from semi-professional team-sport athletes through elite international race-walkers), and given that only k = 5 study-level entries per domain are available (k = 3 with quantitative data in the lactate domain), the pooled effect sizes reported in Section 4.3 should, therefore, be interpreted as exploratory rather than confirmatory. We did not undertake further subgroup analyses (e.g., by individual outcome-measure type, by depth of carbohydrate restriction, or by athlete sub-population) within each domain, because the resulting per-subgroup sample sizes (k ≤ 2 per cell in most cases) would not have supported meaningful pooled inference and would have risked over interpretation of essentially single-study estimates. The pooled point estimates, prediction intervals, and the differential heterogeneity pattern across domains are accordingly intended to identify the direction of effects and to flag where between-study variability is large enough to preclude a single summary recommendation, rather than to provide definitive numerical estimates of the magnitude of effect for any specific anaerobic outcome.

### 5.7. Publication Bias and Quality Considerations

Publication bias assessment revealed minimal evidence of selective reporting in the anaerobic performance literature. Egger’s regression test was non-significant for both the anaerobic power domain (*p* = 0.42) and the RSA domain (*p* = 0.18), and trim-and-fill analysis identified zero missing studies for the power domain. The universal concordance of all three studies measuring blood lactate concentration showing significant reductions (all *p* < 0.001) represents particularly strong evidence against selective reporting bias for this metabolic outcome, as this outcome shows no variability that selective publication could suppress.

The high methodological quality of the included studies (mean NOS = 7.2 ± 0.8 across 13 unique studies; 92.3% scoring ≥ 7), with successful matching or statistical control of confounding variables in 100% of studies (all studies achieving at least one star on the NOS comparability item) and objective, validated outcome measurement in 100% of studies, indicates that quality differences did not systematically bias conclusions. Quality-stratified sensitivity analyses demonstrated that high-quality and moderate-quality studies showed concordant findings across all three anaerobic performance domains (power, RSA, blood lactate), arguing against quality-related bias in effect estimates.

It is also noteworthy that studies reporting performance impairments were usually conducted on elite or highly trained athletes, while studies finding no effect or a performance enhancing effect were typically conducted on moderately trained athletes. This pattern may reflect a difference in reliance on glycolytic pathways between the two groups, rather than publication bias. Elite performers, who operate closer to their physiological limits, may be more susceptible to disruptions in high-intensity metabolic pathways. This highlights the importance of interpreting results in the context of a specific population.

### 5.8. Practical Implications for Athletes and Coaches

From a practical perspective, the findings of the present review suggest that athletes and practitioners should carefully consider the metabolic demands of their sport when implementing carbohydrate-restricted dietary strategies. While carbohydrate-restricted diets may promote metabolic adaptations that support prolonged submaximal exercise, many competitive sports require frequent high-intensity efforts that rely heavily on carbohydrate metabolism. In such contexts, strict carbohydrate restriction may compromise the ability to sustain repeated high-intensity efforts. Therefore, rather than adopting chronically low-carbohydrate dietary patterns, a carbohydrate-periodized approach, where carbohydrate availability is adjusted according to training intensity and competition demands, may represent a more effective strategy for balancing metabolic adaptations with performance requirements.

## 6. Conclusions

This systematic review and meta-analysis synthesizing evidence from 15 studies (identified from 49 retrieved records), examining low-carbohydrate and ketogenic diet effects on anaerobic performance outcomes in trained athletes, provides comprehensive evidence for several fundamental conclusions regarding anaerobic power capacity, repeated sprint ability, blood lactate responses, and temporal adaptation patterns.

### 6.1. Primary Findings

Anaerobic performance outcomes related to single, short-duration efforts appear relatively preserved under carbohydrate-restricted conditions. Measures of anaerobic power output (e.g., Wingate testing, vertical jump, strength assessments) were maintained or improved across the five studies examining these variables, with no study reporting statistically significant impairment. However, these findings should be interpreted cautiously due to the limited number of studies and do not preclude impairments in other domains of anaerobic performance, particularly repeated high-intensity exercise.

Repeated sprint ability showed no significant difference in three of five studies, while two studies reported transient decrements during high-intensity interval performance, suggesting that this domain may be particularly susceptible to carbohydrate restriction.

Blood lactate concentrations demonstrated consistent reductions across the three studies reporting this variable (all *p* < 0.001). These reductions should be interpreted as a consequence of reduced glycolytic flux due to limited carbohydrate availability, rather than a favorable metabolic adaptation. While this reflects altered substrate utilization during exercise, it may also indicate a reduced capacity to sustain high-intensity efforts.

### 6.2. Quality of Evidence and Methodological Consistency

The evidence base for anaerobic performance outcomes demonstrates generally good methodological quality. The mean Newcastle–Ottawa Scale score of 7.2 ± 0.8 across the 13 unique included studies (92.3% scoring ≥ 7) indicates that most investigations employed robust designs with appropriate participant selection, comparability, and outcome assessment. Quality-stratified sensitivity analyses revealed consistent findings between high- and moderate-quality studies across all three anaerobic performance domains, suggesting that methodological differences did not materially influence the direction of results.

Assessment of publication bias did not indicate clear evidence of systematic selective reporting. Egger’s regression test was non-significant for both the power domain (*p* = 0.42) and repeated sprint ability (RSA) domain (*p* = 0.18), and trim-and-fill analysis did not identify missing studies for the power outcome. However, interpretation of reporting bias in the metabolic domain is constrained by incomplete outcome reporting, as only three of five studies assessing blood lactate provided extractable quantitative data. Although all three reported significantly lower lactate concentrations under low-carbohydrate or ketogenic conditions (*p* < 0.001), the absence of data from the remaining studies limits the ability to definitively exclude reporting bias.

Notwithstanding these limitations, the consistency of preserved anaerobic power across five independent investigations conducted in diverse athletic populations and research settings supports the robustness of this primary outcome.

### 6.3. Generalizability and Population Representation

Generalizability strengths of the anaerobic performance evidence base include diverse intervention durations (2 days to 12 weeks), multiple athlete populations (endurance athletes, elite racewalkers, strength and power athletes, combat sport athletes, team sport athletes), and measurement across three distinct anaerobic outcome domains (maximal power, repeated sprint ability, blood lactate). Limitations include relative underrepresentation of strength and power athletes (contributing only 1 of 13 unique studies/1 of 15 study-level entries: Greene et al. [34]), overrepresentation of endurance-trained populations, and restriction to English-language publications potentially missing relevant non-English literature. The predominance of cross-over designs (10 of 15 study-level entries, 66.7%; from 9 unique cross-over studies, 69.2%) provides robust within-subject comparisons; however, the parallel-group/RCT designs (5 of 15 entries, 33.3%; from 4 unique parallel-arm studies, 30.8%) showed concordant directional findings within their respective population types, suggesting that carryover effects did not systematically distort anaerobic performance conclusions.

### 6.4. Integrated Conclusions

In conclusion, this systematic review and meta-analysis indicates that low-carbohydrate and ketogenic dietary interventions do not appear to substantially impair single, short-duration anaerobic performance, but may negatively affect repeated high-intensity performance depending on the exercise context in trained athletes. Maximal anaerobic power output, assessed via Wingate testing, countermovement jump, and one-repetition maximum strength, was maintained or improved across the five studies examining these variables, with a small, non-significant pooled effect (pooled d = +0.29, 95% CI: −0.08 to +0.66).

In contrast, repeated sprint ability showed no significant difference in three of five studies, while two studies reported transient impairments during high-intensity interval performance, with a small, non-significant negative pooled effect (pooled d = −0.33, 95% CI: −0.80 to +0.14). These findings suggest that this domain may be particularly susceptible to carbohydrate restriction under specific conditions, especially during short-term adaptation phases.

Blood lactate concentrations were consistently and significantly lower across the three studies reporting this outcome (pooled d = −0.89, 95% CI: −1.20 to −0.58; all *p* < 0.001). These reductions should be interpreted as a consequence of reduced glycolytic flux due to limited carbohydrate availability rather than a favorable metabolic adaptation and may reflect a reduced capacity to sustain high-intensity efforts.

Some evidence suggests that longer-term dietary adaptation (>42 days) may attenuate these effects; however, the limited number of studies and variability in study design warrant cautious interpretation. Overall, the available evidence demonstrates moderate methodological quality and generally consistent findings across studies, supporting a domain-specific effect of carbohydrate-restricted diets on anaerobic performance in trained athletes. The pooled effect-size estimates reported in this synthesis should be interpreted as exploratory rather than confirmatory, given the small number of studies per meta-analytic domain (k = 3–5 entries), the substantial heterogeneity in intervention protocols and outcome measures, and the wide 95% prediction intervals that cross zero in two of the three primary domains.

## 7. Implications for Athletes, Coaches, and Practitioners

The findings of the present systematic review and meta-analysis have several practical implications for athletes, coaches, and sports nutrition practitioners. First, the available evidence suggests that carbohydrate-restricted diets do not universally impair anaerobic performance in trained athletes, particularly during single, short-duration maximal efforts that rely primarily on the ATP–phosphocreatine system. In several studies, peak power output during short sprint tests was maintained or even slightly improved following carbohydrate restriction, potentially reflecting reductions in body mass or improvements in metabolic efficiency.

However, the results also indicate that carbohydrate restriction may negatively affect performance during repeated high-intensity efforts, which depend more heavily on glycolytic metabolism and sufficient intramuscular glycogen availability. Activities such as repeated sprint bouts, high-intensity interval training, or team sport actions require rapid ATP turnover through glycolysis, and reduced glycogen stores may compromise the capacity to sustain these efforts over multiple repetitions.

Therefore, athletes participating in sports characterized by repeated high-intensity efforts, such as team sports, combat sports, and numerous endurance sport disciplines involving surges and sprint finishes, should carefully consider the potential performance implications of chronic carbohydrate restriction. In such contexts, strict ketogenic or low-carbohydrate dietary strategies may not be optimal for supporting maximal performance.

A more flexible nutritional strategy, such as carbohydrate periodization, may provide a more effective balance between metabolic adaptation and performance support. Adjusting carbohydrate intake according to training intensity, competition demands, and recovery needs allows athletes to benefit from metabolic adaptations associated with lower carbohydrate availability while still maintaining sufficient glycogen stores for high-intensity exercise. Consequently, individualized nutritional strategies tailored to the specific physiological demands of a given sport and athlete may represent the most practical approach for optimizing performance.

## 8. Future Research Directions

Despite the growing number of studies examining the effects of low-carbohydrate and ketogenic diets on athletic performance, several important gaps remain in the current evidence base. First of all, many of the available studies involve relatively small sample sizes and short intervention durations, limiting the ability to draw definitive conclusions regarding long-term adaptations to carbohydrate-restricted diets in athletic populations. Future research should prioritize larger randomized controlled trials with longer intervention periods to better understand the chronic effects of these dietary strategies on performance outcomes.

Secondly, the majority of studies included in the present review focused on endurance-trained athletes rather than athletes from sports where anaerobic performance is the primary determinant of success. Investigations involving strength and power athletes, team sport athletes, and sprint specialists would provide valuable insights into the applicability of carbohydrate-restricted diets in these populations.

Third of all, future studies should aim to better characterize the relationship between dietary carbohydrate availability, intramuscular glycogen distribution, and specific components of anaerobic performance, including peak power, repeated sprint ability, and fatigue resistance. Advances in muscle glycogen imaging techniques and metabolomic analysis may help clarify how carbohydrate restriction affects metabolic processes within different muscle fiber types and subcellular compartments Some metabolic and performance aspects of low carbohydrate diets could be explained by assessing the activity of enzymes involved in anaerobic glycolysis, particularly hexokinase, phosphofructokinase, glyceraldehyde-3-phosphate dehydrogenase, pyruvate kinase, and lactate dehydrogenase. Their influence on exercise capacity during prolonged sprints lasting 40–60 s could be evaluated.

Additionally, future research should explore the interaction between carbohydrate restriction and training periodization strategies. Understanding how targeted carbohydrate availability around high-intensity training sessions influences adaptation, recovery, and performance may help identify nutritional strategies that optimize both metabolic flexibility and competitive performance.

Finally, further investigation into individual variability in response to carbohydrate-restricted diets is warranted. Factors such as training status, metabolic phenotype, genetic differences, and sport-specific demands may influence the extent to which athletes respond favorably or unfavorably to low-carbohydrate nutritional strategies.

## 9. Final Summary

In summary, the present systematic review and meta-analysis examined the effects of low-carbohydrate and ketogenic diets on anaerobic performance outcomes in trained athletes. The available evidence suggests that while short-duration maximal efforts are generally preserved under carbohydrate-restricted conditions, performance during repeated high-intensity efforts may be negatively affected. These findings likely reflect the differing metabolic demands of anaerobic exercise, with single explosive efforts relying primarily on phosphocreatine metabolism, whereas repeated high-intensity actions require substantial glycolytic ATP production supported by adequate glycogen availability.

Overall, the results highlight that the effects of carbohydrate-restricted diets on athletic performance are highly dependent on the specific physiological demands of the exercise task. While low-carbohydrate strategies may provide certain metabolic advantages in specific contexts, they may also introduce limitations when rapid and repeated high-intensity energy production is required.

Consequently, nutritional strategies for athletes should consider the interaction between dietary carbohydrate availability, training demands, and competition requirements. Individualized approaches that integrate carbohydrate periodization may offer a practical framework for balancing metabolic adaptations with the performance demands of high-intensity sport.

Overall, the results of this systematic review and meta-analysis indicate that carbohydrate-restricted dietary strategies exert domain-specific effects on anaerobic performance in trained athletes. While single, short-duration maximal efforts appear largely preserved under low-carbohydrate or ketogenic conditions, performance during repeated high-intensity efforts may be negatively affected, likely due to reduced intramuscular glycogen availability and limitations in glycolytic ATP production. These findings highlight the importance of considering the specific metabolic demands of the exercise task when evaluating the suitability of carbohydrate-restricted diets for athletic performance. Consequently, nutritional strategies for athletes should be individualized and aligned with the intensity and structure of training and competition, with carbohydrate periodization representing a potentially effective approach for balancing metabolic adaptations with the requirements of high-intensity exercise.

## Figures and Tables

**Table 1:** Distribution of study design methodologies (*n* = 13 unique studies; 15 study-level entries).

Study Design Category	Unique Studies (*n* = 13)		Study-Level Entries (*n* = 15)	
	Number	%	Number	%
Randomized Cross-over/ Repeated-Measures Cross-over	9	69.2	10	66.7
Parallel Group/Randomized Controlled Trial	4	30.8	5	33.3
Cross-sectional	0	0.0	0	0.0
Total	13	100.0	15	100.0

Note. Two studies (Prins et al. [27] and McKay et al. [26]) each contributed to two of the three meta-analytic domains, so the entry total (15) exceeds the unique-study total (13). See Supplementary Table S2 for study-specific design characteristics.

**Table 2:** Distribution of intervention duration categories (*n* = 13 unique studies; 15 study-level entries).

Intervention Duration	Unique Studies (*n* = 13)		Study-Level Entries (*n* = 15)	
	Number	%	Number	%
≤7 days	4	30.8	4	26.7
8–14 days	1	7.7	1	6.7
15–31 days	3	23.1	5	33.3
≥6 weeks (6–12 weeks)	5	38.5	5	33.3
>3 months	0	0.0	0	0.0
Not specified	0	0.0	0	0.0
Total	13	100.0	15	100.0

Note. Both Prins et al. [27] (31 days) and McKay et al. [26] (3 weeks = 21 days) fall within the 15–31 days bucket and each contribute to two meta-analytic domains; this is why the 15–31 days bucket reduces from 5 entries to 3 unique studies while other buckets are unchanged.

**Table 3:** Distribution of performance outcome measures across study-level entries (*n* = 15 entries from 13 unique studies).

Performance Measure Category	Number of Study-Level Entries	Percentage (%)
Maximal anaerobic power outputs (Wingate test, CMJ, sprint power, 1RM)	5	33.3
Repeated sprint ability and high-intensity interval performance	5	33.3
Blood lactate concentration (metabolic anaerobic marker)	5	33.3
Total	15	100.0

Note. Each entry represents a study × meta-analytic domain pairing; counts are presented at the entry level because each domain (Power, RSA, Lactate) constitutes a separate pooled meta-analysis. Two studies (Prins et al. [27] and McKay et al. [26]) contribute to both the RSA and Lactate domains, accounting for the difference between 15 entries and 13 unique studies. Complete information on performance measures is provided in Supplementary Tables S1 and S4.

**Table 4:** Anaerobic performance meta-analysis—Wingate test outcomes.

Study	Year	Design	N	Population	Intervention	Duration	Variable	Effect	Control Comparison	*p*-Value	Category
[39]	2021	Parallel RCT	16	Soccer players	KD	30 days	Countermovement jump	Improved	Western diet: no improvement	0.0021	↑
[23]	2018	Crossover	47	Mixed trained	KD	12 days	Sprint peak power	Increased	High-carb: decreased	0.025	↑
[36]	2013	Crossover	31	Mixed (M/W)	LCD	7 days	Wingate, 1RM, jump	Maintained	Habitual: maintained	NS	—
[34]	2018	Crossover	14	Strength/Power	KD	3 months	1RM various lifts	No difference	Usual: no difference	NS	—
[30]	1994	Crossover	5	Cyclists	LCD	2 weeks	Wingate power	No difference	High-carb: no difference	NS	—

Symbol legend: ↑ = improved/increased performance; — = maintained/no significant difference; KD—ketogenic diet; LCD—low carbohydrate diet.

**Table 5:** Anaerobic performance meta-analysis—repeated sprint ability (5 studies).

Study	Year	Design	N	Population	Intervention	Duration	Variable	Effect	Control Comparison	*p*-Value	Category
[27]	2023b	Crossover	10	Runners	LCD	6 weeks	6 × 800 m runs	Equivalent	High-carb: equivalent	NS	—
[35]	2023	Crossover	12	Taekwondo	LCD/Moderate carbohydrates	7 days	Repeated sprint ability	No difference	Mixed control: no difference	NS	—
[37]	2023	Crossover	9	Runners	LCD	2 days	Time trials 50–3000 m	No difference	High-carb: no difference	NS	—
[26]	2023	Parallel	21	Racewalkers	KD	3 weeks	Interval speed	8% slower	High-carb: faster	0.001	↓
[32]	2022	Parallel	28	Racewalkers	KD	3 weeks	Interval speed	8% slower	High-carb: faster	<0.001	↓

Symbol legend: ↓ = deteriorated/decreased performance; — = maintained/no significant difference; KD—ketogenic diet; LCD—low carbohydrate diet.

**Table 6:** Anaerobic performance meta-analysis—lactate concentration and acid-base balance (5 studies).

Study	Year	Design	N	Population	Intervention	Duration	Variable	Effect	Control Comparison	*p*-Value	Category
[31]	2024	Parallel RCT	65	Moderately trained	KD	10 weeks	Blood lactate	Decreased	High-GI: unchanged	<0.001	↓
[26]	2023	Parallel	21	Racewalkers	KD	3 weeks	Lactate	Lower	High-carb: higher	<0.001	↓
[33]	2020	Parallel	28	Elite racewalkers	LCD periodized	25 days	Lactate	Lower	High-carb: higher	<0.001	↓
[38]	2018	Crossover	8	Cyclists	LCD	5 days	Blood lactate	Not reported	High-carb: not reported	NR	?
[27]	2023b	Crossover	10	Runners	LCD	6 weeks	Lactate	Not reported	High-carb: not reported	NR	?

Symbol legend: ↓ = decreased/lower lactate; ? = not reported; KD—ketogenic diet; LCD—low carbohydrate diet.

## Data Availability

Data supporting the findings of this systematic review and meta-analysis are available within the article and its Supplementary Materials. The extracted dataset (study characteristics, Newcastle–Ottawa Scale scores, and effect-size data entering the meta-analyses) and the analysis code are available from the corresponding author on reasonable request (a.zajac@awf.katowice.pl).

## Supplementary Materials

The following supporting information can be downloaded at: https://www.mdpi.com/article/10.3390/nu18101589/s1. Table S1. PRISMA 2020 checklist; Table S2. Characteristics of all 13 unique studies (15 study-level entries) included in the systematic review and meta-analysis; Table S3. Full electronic database search strategy (PubMed, with syntactic adaptations for Scopus, Web of Science, SPORTDiscus and Cochrane CENTRAL); Table S4. Newcastle–Ottawa Scale individual item-level quality assessment for each included study (S1–S4, C1, O1–O3); Table S5. Field-level breakdown of LLM-assisted vs. human data-extraction discrepancies, resolution mechanism, and inter-rater agreement statistics [28].

## Abbreviations

The following abbreviations are used in this manuscript:

CHO	Carbohydrate
KD	Ketogenic diet
LC	Low carbohydrate
LCHF	Low-carbohydrate high-fat
1RM	One-repetition maximum
CMJ	Countermovement jump
RSA	Repeated sprint ability
TT	Time trial
VO_2_max	Maximal oxygen uptake
NOS	Newcastle–Ottawa Scale
NR	Not reported
RCT	Randomized controlled trial
