# Supplementary material for: Effects of Low-Carbohydrate and Ketogenic Diets on Anaerobic Performance in Competitive Athletes: A Systematic Review and Meta-Analysis
Source: Nutrients. 2026 May 16;18(10):1589. doi: 10.3390/nu18101589 (PMC13209462; doi:10.3390/nu18101589)
Supplement: Supplementary file 1 [file nutrients-18-01589-s001.zip › nutrients-4276806-supplementary.pdf]

Supplementary Tables

Table S1. PRISMA 2020 checklist

Item #	Item	Checklist item (PRISMA 2020)	Location in manuscript
**TITLE**			
1	Title	Identify the report as a systematic review.	Title page: "Effects of Low-Carbohydrate and Ketogenic Diets on Anaerobic Performance in Competitive Athletes: A Systematic Review and Meta-Analysis"
**ABSTRACT**			
2	Abstract	See the PRISMA 2020 for Abstracts checklist.	Abstract (Background/Objectives, Methods, Results, Conclusions)
**INTRODUCTION**			
3	Rationale	Describe the rationale for the review in the context of existing knowledge.	Section 1 (Introduction), paragraphs 1–5
4	Objectives	Provide an explicit statement of the objective(s) or question(s) the review addresses.	Section 1, final paragraph
**METHODS**			
5	Eligibility criteria	Specify the inclusion and exclusion criteria and how studies were grouped for the syntheses.	Section 2.2 (PICOS criteria 2.2.1–2.2.5)
6	Information sources	Specify all databases, registers, websites, organizations, reference lists and other sources searched; specify date last searched.	Section 2.1; final search October 2025
7	Search strategy	Present full search strategies for all databases, registers and websites, including any filters and limits.	Section 2.1 and Supplementary Table S2
8	Selection process	Specify methods used to decide whether a study met inclusion criteria, including number of reviewers, whether they worked independently, and any automation tools used.	Section 2.3 (two reviewers working independently; κ = 0.78; Elicit used for initial retrieval)
9	Data collection process	Specify methods used to collect data from reports, including number of reviewers, whether they worked independently, and any processes for obtaining or confirming data from study investigators.	Section 2.4 (two independent reviewers; LLM-assisted extraction with human verification)
10a	Data items – outcomes	List and define all outcomes for which data were sought.	Section 2.2.4 and 2.4(6)–(7)

Item #	Item	Checklist item (PRISMA 2020)	Location in manuscript
10b	Data items – other variables	List and define all other variables for which data were sought (e.g., population, intervention, funding sources).	Section 2.4 (items 1–9, including funding sources of the included studies and methodological quality)
11	Study risk of bias assessment	Specify the methods used to assess risk of bias in the included studies, including details of the tool(s) used, number of reviewers, and whether they worked independently.	Section 2.4 (Newcastle–Ottawa Scale; two independent reviewers); rationale for NOS over RoB2 explicitly stated
12	Effect measures	Specify the effect measure(s) used in the synthesis or presentation of results.	Section 2.5.1 (standardized mean difference, Cohen's d; odds ratios with 95% CI)
13a	Synthesis methods – eligibility for synthesis	Describe the processes used to decide which studies were eligible for each synthesis.	Section 2.5 and 2.3
13b	Synthesis methods – preparation of data	Describe any methods required to prepare the data for presentation or synthesis.	Section 2.5.1
13c	Synthesis methods – tabulation/visualisation	Describe any methods used to tabulate or visually display results.	Section 3 (forest plots, Tables 1–3)
13d	Synthesis methods – statistical synthesis	Describe methods used to synthesize results and provide a rationale; identify the model used and methods to identify heterogeneity.	Section 2.5.1–2.5.2 (random-effects DerSimonian–Laird where $I^2 \geq 25\%$; Cochran's Q; I^2)
13e	Synthesis methods – heterogeneity exploration	Describe any methods used to explore possible causes of heterogeneity among study results.	Section 2.5.3 (subgroup and meta-regression analyses)
13f	Synthesis methods – sensitivity analyses	Describe any sensitivity analyses conducted to assess robustness of the synthesized results.	Section 4.3
14	Reporting bias assessment	Describe any methods used to assess risk of bias due to missing results in a synthesis (arising from reporting biases).	Section 2.5.4 (Egger's test; Begg's test; trim-and-fill; funnel plots)
15	Certainty assessment	Describe any methods used to assess certainty (or confidence) in the body of evidence for an outcome.	Section 5.7 (quality-stratified sensitivity analyses; NOS-based evidence grading)
**RESULTS**			
16a	Study selection – results	Describe the results of the search and selection process; report the number of records identified, included and excluded, and the reasons for exclusions.	Section 2.3 and Figure S1 (PRISMA flow diagram)

Item #	Item	Checklist item (PRISMA 2020)	Location in manuscript
16b	Study selection – list of excluded studies	Cite studies that might appear to meet the inclusion criteria, but which were excluded, and explain why they were excluded.	Reasons for full-text exclusions summarized in Figure S1
17	Study characteristics	Cite each included study and present its characteristics.	Section 3, Tables 1–3
18	Risk of bias in studies	Present assessments of risk of bias for each included study.	Section 4.2 and Supplementary Table S3 (NOS scores per study)
19	Results of individual studies	For all outcomes, present, for each study: summary statistics for each group and effect estimate with its precision.	Tables 1–3 and Figures 2–4
20a	Results of syntheses – brief summary	For each synthesis, briefly summarize the characteristics and risk of bias among contributing studies.	Section 4.1
20b	Results of syntheses – statistical results	Present results of all statistical syntheses; if meta-analysis was performed, present for each the summary estimate and its precision (e.g., 95% CI) and measures of statistical heterogeneity.	Section 4.1; pooled Cohen's d with 95% CI and I^2 for each domain
20c	Results of syntheses – heterogeneity investigation	Present results of all investigations of possible causes of heterogeneity among study results.	Section 4.1 and 4.3
20d	Results of syntheses – sensitivity analyses	Present results of all sensitivity analyses conducted to assess the robustness of the synthesized results.	Section 4.3 (leave-one-out analyses)
21	Reporting biases	Present assessments of risk of bias due to missing results for each synthesis assessed.	Section 4.2 and 5.7 (Egger's $p = 0.42$ for power, $p = 0.18$ for RSA; trim-and-fill: 0 missing)
22	Certainty of evidence	Present assessments of certainty (or confidence) in the body of evidence for each outcome assessed.	Section 5.7 (mean NOS 7.2 ± 0.8; quality-stratified sensitivity analyses concordant)
**DISCUSSION**			
23a	Discussion – interpretation	Provide a general interpretation of the results in the context of other evidence.	Section 5 (Discussion), subsections 5.1–5.6
23b	Discussion – limitations of evidence	Discuss any limitations of the evidence included in the review.	Section 5.7
23c	Discussion – limitations of review processes	Discuss any limitations of the review processes used.	Section 5.7 and 6.3
23d	Discussion – implications	Discuss implications of the results for practice, policy and future research.	Sections 5.8, 7 and 8

Item #	Item	Checklist item (PRISMA 2020)	Location in manuscript
**OTHER INFORMATION**			
24a	Registration and protocol – registration	Provide registration information for the review, including register name and registration number, or state that the review was not registered.	Section 2.1 (PROSPERO CRD420261277181; broader protocol covering aerobic + anaerobic outcomes)
24b	Registration and protocol – protocol access	Indicate where the review protocol can be accessed, or state that a protocol was not prepared.	PROSPERO record CRD420261277181
24c	Registration and protocol – amendments	Describe and explain any amendments to information provided at registration or in the protocol.	None beyond the bifurcation of the synthesis into companion aerobic and anaerobic manuscripts (pre-planned)
25	Support	Describe sources of financial or non-financial support for the review, and the role of the funders or sponsors in the review.	Funding statement ("This research received no external funding")
26	Competing interests	Declare any competing interests of review authors.	Conflicts of Interest statement
27	Availability of data, code and other materials	Report which of the following are publicly available and where they can be found: template data collection forms; data extracted from included studies; data used for all analyses; analytic code; any other materials used in the review.	Data Availability Statement; extracted data available from the corresponding author on reasonable request

Supplementary Table S2. Characteristics of all 13 unique studies (15 study-level entries) included in the systematic review and meta-analysis.

No.	Reference	Study Design	N	Population	Intervention Diet / CHO Restriction	Duration	Control Condition	Performance Outcomes Measured	Key Results (Primary Anaerobic Outcomes)	Main Limitations	NOS Score	Meta-Analysis Domain
1	Antonio Paoli et al. (2021)	Parallel group / RCT	16	Semi-professional male soccer players, age = 25.5 ± 2.8 years, height = 179.0 ±...	Ketogenic diet (KD), <30 g/day CHO, ~65–70% fat, 1.8 g/kg body protein	NR	Western diet (WD)	Aerobic: YYIR1; Anaerobic: CMJ; Metabolic: REE, RER	Body fat: KD −1.55 kg, WD −0.92 kg ($p = 0.0359$); VAT decreased; CMJ unchanged	Potential DXA errors; small n; limited measurements	7/9	Power (Table 1)
2	McSwiney et al. (2018)	Crossover	47	Male endurance-trained athletes, 18–40 yr, >2 yr training experience	LCKD, >75% fat, 10–15% protein, <50 g/day CHO, 12 weeks	12 weeks	High-CHO	Aerobic: VO_2max, 100 km TT; Anaerobic: SS sprint peak power, CPT peak power; Metabolic: fat oxidation, lactate	Body mass −5.9 kg LCKD; βHB rose; 100 km TT slower; sprint power maintained	Non-randomized; high dropout; small n	7/9	Power (Table 1)
3	Sawyer et al. (2013)	Crossover	31	Trained men and women	Carbohydrate-restricted (5.4% energy	7 days	Habitual diet	Anaerobic: handgrip, vertical jump,	Performance largely maintained across measures	Small n; short duration; mixed sex	7/9	Power (Table 1)

No.	Reference	Study Design	N	Population	Intervention Diet / CHO Restriction	Duration	Control Condition	Performance Outcomes Measured	Key Results (Primary Anaerobic Outcomes)	Main Limitations	NOS Score	Meta-Analysis Domain
					CHO, 53.6% fat)			1RM bench/squat, max-rep bench, 30 s Wingate				
4	Greene et al. (2018)	Crossover	14	Intermediate-to-elite powerlifters and Olympic weightlifters	LCKD, ≤50 g or ≤10% CHO, 70% fat, 20% protein, 3 months	3 months	High-CHO (usual diet)	Anaerobic: 1RM snatch, clean & jerk, squat, bench, deadlift; Metabolic: RMR, RQ	Body mass and lean mass decreased; lifting performance maintained	Insufficient n for sex effects; ad libitum intake variability	8/9	Power (Table 1)
5	Lambert et al. (1994)	Crossover	5	Trained cyclists	High-fat (70% fat, 7% CHO)	2 weeks	High-CHO (2 weeks)	Aerobic: VO_2max, time to exhaustion; Anaerobic: Wingate; Metabolic: RER, CHO oxidation	TTE not different; muscle glycogen lower on HIGH-FAT	Very small n (n = 5); narrow exercise range	6/9	Power (Table 1)

No.	Reference	Study Design	N	Population	Intervention Diet / CHO Restriction	Duration	Control Condition	Performance Outcomes Measured	Key Results (Primary Anaerobic Outcomes)	Main Limitations	NOS Score	Meta-Analysis Domain
6	Prins et al. (2023b)	Crossover	10	Male competitive distance runners (30–50 yr)	LCHF; <50 g/day CHO; 75–80% fat	31 days	31 days HCLF	Aerobic: VO_2max, HR, RER; Anaerobic: 6 × 800 m repeated sprints; Metabolic: lactate	Equivalent high-intensity performance; record peak fat oxidation 1.58 g/min on LCHF	Generalizability to females limited; methodological constraints	8/9	RSA (Table 2) + Lactate (Table 3)
7	Chin Hsu et al. (2023)	Crossover	12	Male taekwondo athletes	Low-carb, 10% energy CHO, 49% fat, 41% protein	7 days	7 days moderate-CHO	Anaerobic: RSA tests, average and peak power	Body mass decreased similarly; RSA average/peak power not different	Small n; short duration; single sport	7/9	RSA (Table 2)
8	Ramonas et al. (2023)	Crossover	9	Male runners, VO_2peak 60.3 ± 3.3 $mL·kg^{-1}·min^{-1}$	Low-carb, <50 g/day CHO	Until completion of performance battery	High-CHO	Aerobic: running economy, critical speed; Anaerobic: TT 50 m–3000 m;	No TT differences ($P > 0.05$); fat oxidation increased on LOW	Effects on training quality; individual variability	7/9	RSA (Table 2)

No.	Reference	Study Design	N	Population	Intervention Diet / CHO Restriction	Duration	Control Condition	Performance Outcomes Measured	Key Results (Primary Anaerobic Outcomes)	Main Limitations	NOS Score	Meta-Analysis Domain
								Metabolic: fat oxidation				
9	Alannah K A McKay et al. (2023)	Parallel group / RCT	NR	Elite male racewalkers; matched on PB, age/training age	LCHF (ketogenic), <50 g/day CHO, 75–80% fat	3 weeks	Same volume as HCHO but varied CHO availability	Aerobic: VO_2max, HR, RER; Metabolic: lactate, glucose, ketones	LCHF training volume and walking speed lower than HCHO/PCHO ($P < .001$)	Possibly inadequate adaptation time; volume confound	8/9	RSA (Table 2) + Lactate (Table 3)
10	Alannah K A McKay et al. (2022)	Crossover	28	Elite male race walkers	LCHF, <50 g/day CHO	3 weeks	3 weeks HCHO/PCHO	Aerobic: normalized speed, HR; Metabolic: post-exercise lactate, ketones	LCHF 8.0–8.2% slower than HCHO/PCHO during intervals ($p < 0.001$)	Limited to elite male race walkers	7/9	RSA (Table 2)
11	Moitzi et al. (2024)	Parallel group / RCT	65	Moderately trained healthy men; 29 ± 4 yr;	LCHF (ketogenic), ≤ 50 g	10 weeks	LOW-GI (50–60% CHO)	Aerobic: VO_2max, TTE, peak running speed;	Fat oxidation rose in LCHF; lactate fell in LOW-GI and LCHF; peak speed	Self-reported diet; potential confounders	8/9	Lactate (Table 3)

No.	Reference	Study Design	N	Population	Intervention Diet / CHO Restriction	Duration	Control Condition	Performance Outcomes Measured	Key Results (Primary Anaerobic Outcomes)	Main Limitations	NOS Score	Meta-Analysis Domain
				VO_2peak 55 ± 8 mL·min^{-1}·kg^{-1}	CHO/day, ≥ 65% fat			Metabolic: lactate, RER	unchanged in LCHF			
12	Burke et al. (2020)	Parallel group / RCT	28	Highly competitive male and female race walkers	LCHF, <50 g/day CHO, 78% energy fat, 2.1 g/kg/day protein	NR	HCHO (60–65% energy CHO)	Aerobic: VO_2max, walking economy; Metabolic: lactate, glucose, βHB, RER	VO_2peak rose in all groups; LCHF fat oxidation ↑; HCHO 10 km performance ↑ 4.8% ($p < 0.001$)	Non-randomized allocation; environmental control limits	9/9	Lactate (Table 3)
13	Leckey et al. (2018)	Crossover	8	Well-trained male cyclists, 25 ± 4 yr	High-fat (HFAT), >65 % fat, <20% CHO	NR	High-CHO (HCHO)	Aerobic: VO_2max, PPO, HR, RER; Anaerobic: TT performance; Metabolic: lactate, glucose	Mitochondrial respiration ↓ in HFAT; fat oxidation ↑; CHO oxidation ↓	Participants not blinded; small biopsy n	7/9	Lactate (Table 3)

Notes: Studies are presented in the order they appear in the three primary meta-analysis tables. Two studies (Prins et al. 2023b and McKay et al. 2023) contributed data to two meta-analysis domains simultaneously, yielding 15 study-level entries from 13 unique primary studies. All NOS scores are on a 0–9 scale; scores ≥ 7 indicate high methodological quality. Key results presented focus on primary anaerobic outcomes; see original publications for full results. Supplementary Table S4 contains individual Newcastle–Ottawa Scale item scores for each study.

nutrients MDPI

Supplementary Table S3. Full electronic database search strategy.

A. PubMed / MEDLINE search strategy (primary database)

Line	Query
#1	"Diet, Ketogenic"[MeSH] OR "Diet, Carbohydrate-Restricted"[MeSH] OR "ketogenic diet"[tiab] OR "keto diet"[tiab] OR "low-carbohydrate diet"[tiab] OR "low carbohydrate"[tiab] OR "low-carb"[tiab] OR "low-CHO"[tiab] OR "LCHF"[tiab] OR "low carbohydrate high fat"[tiab]
#2	"Athletic Performance"[MeSH] OR "Exercise"[MeSH] OR "Sports"[MeSH] OR "athletic performance"[tiab] OR "exercise performance"[tiab] OR "sprint"[tiab] OR "Wingate"[tiab] OR "peak power"[tiab] OR "mean power"[tiab] OR "anaerobic power"[tiab] OR "anaerobic capacity"[tiab] OR "repeated sprint ability"[tiab] OR RSA[tiab] OR "countermovement jump"[tiab] OR CMJ[tiab] OR "one-repetition maximum"[tiab] OR "1RM"[tiab] OR "blood lactate"[tiab]
#3	"Athletes"[MeSH] OR athlete*[tiab] OR "trained"[tiab] OR "well-trained"[tiab] OR "highly trained"[tiab] OR "elite"[tiab] OR "competitive"[tiab]
#4	"Randomized Controlled Trial"[pt] OR "Clinical Trial"[pt] OR "Cross-Over Studies"[MeSH] OR randomi*[tiab] OR crossover[tiab] OR "cross-over"[tiab] OR "controlled trial"[tiab] OR intervention*[tiab]
#5	#1 AND #2 AND #3 AND #4
Filters	English; Humans
Date of search	October 2025
Records retrieved (PubMed)	See Section 2.1 and Figure 1

B. Syntactic adaptations for the remaining four databases

Database	Syntactic adaptation
Scopus	TITLE-ABS-KEY("ketogenic diet" OR "low-carbohydrate" OR LCHF OR "low-CHO") AND TITLE-ABS-KEY("athletic performance" OR Wingate OR sprint OR "repeated sprint" OR "peak power" OR "mean power" OR "anaerobic") AND TITLE-ABS-KEY(athlete* OR trained OR elite OR competitive) AND TITLE-ABS-KEY(random* OR crossover OR "controlled trial" OR intervention*) AND LANGUAGE(English)
Web of Science	TS=("ketogenic diet" OR "low-carbohydrate" OR LCHF OR "low-CHO") AND TS=("athletic performance" OR Wingate OR sprint OR "repeated sprint" OR "peak power" OR "mean power" OR anaerobic) AND TS=(athlete* OR trained OR elite OR competitive) AND TS=(random* OR crossover OR "controlled trial" OR intervention*); Languages: English
SPORTDiscus (EBSCO)	(AB ("ketogenic diet" OR "low-carbohydrate" OR LCHF) OR TI ("ketogenic diet" OR "low-carbohydrate" OR LCHF)) AND (AB (Wingate OR sprint OR "peak power" OR "anaerobic" OR RSA) OR TI (Wingate OR sprint OR "peak power" OR "anaerobic" OR RSA)) AND (AB (athlete* OR trained OR elite) OR TI (athlete* OR trained OR elite)) AND (AB (random*

Database	Syntactic adaptation
	OR crossover OR intervention*) OR TI (random* OR crossover OR intervention*)); Language: English
Cochrane CENTRAL	("ketogenic diet" OR "low-carbohydrate" OR LCHF OR "low-CHO"):ti,ab,kw AND (Wingate OR sprint OR "peak power" OR anaerobic OR "repeated sprint"):ti,ab,kw AND (athlete* OR trained OR elite):ti,ab,kw

C. Grey literature and supplementary sources

Source	Approach
bioRxiv / medRxiv	Screened via Elicit semantic search across Semantic Scholar and OpenAlex (500-record cap). Preprints identified during screening were included only if peer-reviewed publication status was achieved by the time of full-text assessment.
Reference lists of included studies and relevant reviews	Manual backward-citation screening performed by two reviewers working independently.

Note. Search strings across the four non-PubMed databases were adapted to preserve semantic equivalence with the PubMed strategy, with field tags (e.g., TITLE-ABS-KEY in Scopus, TS in Web of Science, AB/TI in SPORTDiscus, ti,ab,kw in Cochrane CENTRAL) and Boolean syntax adjusted to match each database's native query language. No changes were required in this table to address Comment 17, as it does not reference study counts.

Supplementary Table S4. Newcastle–Ottawa Scale item-level quality assessment for each included study.

No.	Reference	Design	S1	S2	S3	S4	C1	O1	O2	O3	Total
1	Paoli et al. (2021)	Parallel RCT	★	★	★	★	★	★	★	—	7/9
2	McSwiney et al. (2018)	Crossover	★	★	★	★	★	★	★	—	7/9
3	Sawyer et al. (2013)	Crossover	★	★	★	—	★	★	★	★	7/9
4	Greene et al. (2018)	Crossover	★	★	★	★	★★	★	★	★	8/9
5	Lambert et al. (1994)	Crossover	★	★	★	—	★	★	★	★	6/9
6	Prins et al. (2023b)	Crossover	★	★	★	★	★★	★	★	★	8/9
7	Hsu et al. (2023)	Crossover	★	★	★	★	★	★	★	—	7/9
8	Ramonas et al. (2023)	Crossover	★	★	★	★	★	★	★	—	7/9
9	McKay et al. (2023)	Parallel RCT	★	★	★	★	★★	★	★	★	8/9
10	McKay et al. (2022)	Crossover	★	★	★	★	★	★	★	—	7/9
11	Moitzi et al. (2024)	Parallel RCT	★	★	★	★	★★	★	★	★	8/9
12	Burke et al. (2020)	Parallel RCT	★	★	★	★	★★	★	★	★	9/9
13	Leckey et al. (2018)	Crossover	★	★	★	★	★	★	★	—	7/9

Note. Individual item-level Newcastle–Ottawa Scale (NOS) scores for each of the 13 unique primary studies (yielding 15 study-level entries in the pooled meta-analyses) are shown above. Each item receives one star (★) when the criterion is met and no star (—) when it is not. The comparability item (C1) may receive up to two stars. Totals (right-hand column) correspond to the scores reported in Section 4.2 of the main manuscript. Studies 6 and 9 (Prins et al. 2023b and McKay et al. 2023) each contributed to two meta-analytic domains, yielding 15 study-table entries from 13 unique primary studies.

Legend – NOS criteria

Item	Domain	NOS criterion (cohort-style adaptation for dietary intervention trials)
S1	Selection	Representativeness of the exposed (intervention) cohort
S2	Selection	Selection of the non-exposed (control) cohort
S3	Selection	Ascertainment of exposure / dietary intervention (documented compliance, ketone or macronutrient verification)
S4	Selection	Demonstration that outcome of interest was not present at the start of study
C1	Comparability	Comparability of cohorts on the basis of the design or analysis (up to 2★: 1★ for training-status matching; +1★ for age / sex / additional confounder control)
O1	Outcome	Assessment of outcome (objective / validated measurement of anaerobic performance)
O2	Outcome	Follow-up long enough for dietary adaptation and outcome manifestation
O3	Outcome	Adequacy of follow-up of cohorts (complete outcome data / low drop-out)

NOS reference: Wells GA, Shea B, O'Connell D, et al. The Newcastle–Ottawa Scale (NOS) for assessing the quality of non-randomized studies in meta-analyses. Ottawa Hospital Research Institute. Adapted for cohort-style assessment of dietary intervention trials in trained athletes; rationale for NOS use (rather than Cochrane RoB 2) is explained in Section 2.4 of the main manuscript.

Supplementary Table S5. Field-level breakdown of LLM-assisted vs. human data-extraction discrepancies, by extraction category, with resolution mechanism and inter-rater agreement statistics for the human-vs-human verification step.

#	Extraction category	Mean LLM-extracted fields per study	Total LLM-extracted fields (× 15 study-level entries)	LLM-vs-human discrepancies (n)	Discrepancy rate (%)	Type of discrepancy
1	Study identification (authors, publication year, study identification number)	3.0	45	0	0.0	—
2	Population characteristics (n total, n per group, age,	5.0	75	1	1.3	Sample-size value: LLM reported a per-arm n as the total study n in a parallel-arm

#	Extraction category	Mean LLM-extracted fields per study	Total LLM-extracted fields (× 15 study-level entries)	LLM-vs-human discrepancies (n)	Discrepancy rate (%)	Type of discrepancy
	Training status, baseline fitness)					Trial with unequally sized groups
3	Intervention characteristics (CHO g·day^{-1}, fat %, protein %, total energy intake, duration)	5.0	75	0	0.0	—
4	Comparison / control diet composition and duration	2.0	30	0	0.0	—
5	Study methodology (washout duration, randomization method, blinding details)	1.0	15	0	0.0	—
6	Performance outcome protocols (test type, intensity, duration, modality, equipment)	3.0	45	0	0.0	—
7	Key results (baseline / post values, effect sizes, p-values, 95% CI bounds)	2.0	30	2	6.7	(a) Effect size: LLM transcribed the lower bound of a 95% CI as the pooled effect-size point estimate (in-text nested format); (b) baseline / post value: LLM returned the pre-intervention baseline rather than the post-intervention outcome (side-by-side pre/post tables without explicit panel labeling)
8	Adaptation timeline (compliance verification, ketone levels)	0.7	10	0	0.0	—
	**Total LLM-extracted fields (categories 1–8)**	**21.7**	**325**	**3**	**0.9**	
9	Study quality / limitations (NOS item-level scoring, generalizability, methodological concerns)	n/a	n/a (human-only)	n/a	n/a	Human reviewers only; no LLM input

Notes. The Elicit research platform (Elicit, Inc.; Elicit Plus subscription, accessed September–October 2025) generated all first-draft extractions for categories 1–8; the underlying large language models were drawn from the GPT-4 family (OpenAI) and the Claude 3 / 3.5 family (Anthropic), with the specific model deployed for any individual extraction selected internally by the Elicit platform and not exposed to or controlled by the user (see Section 2.4 of the main manuscript). Categorical classifications used downstream in the

synthesis (study design, dietary type, athlete population, performance-domain assignment) and the NOS item-level quality assessment were extracted by the human reviewers without any LLM input. All Elicit-generated extractions were treated as draft annotations and re-verified independently against the original publications by two reviewers working independently and blinded to each other's annotations; the human extraction was taken as ground truth in every case of LLM-vs-human disagreement. All three identified LLM-vs-human discrepancies were resolved by consensus discussion involving a third reviewer, with the original full-text publication serving as the adjudication source. Inter-rater agreement between the two human reviewers across all 325 LLM-extracted fields: raw agreement = 99.1% (322 / 325 fields concordant prior to adjudication); Cohen's κ = 0.94 (95% CI: 0.85–1.00), corresponding to *"almost perfect"* agreement per the Landis & Koch (1977) interpretive scale. The three fields requiring inter-reviewer adjudication corresponded one-to-one with the three LLM-vs-human discrepancies listed above. *Reference.* Landis, J.R.; Koch, G.G. The measurement of observer agreement for categorical data. *Biometrics* 1977, *33*, 159–174. doi:10.2307/2529310.
